# Jaboticaba peel extract limits castration-resistant prostate cancer aggressiveness by counteracting EMT through steroid hormone and TGF-β signaling modulation

**DOI:** 10.1007/s10735-026-10851-x

**Published:** 2026-06-22

**Authors:** Fabiana Regina Schievano, Jaqueline de Souza Gianchetto, Felipe Rabelo Santos, Mário Roberto Maróstica Júnior, Valéria Helena Alves Cagnon, Fabio Montico

**Affiliations:** 1 Department of Structural and Functional Biology, Institute of Biology, University of Campinas (UNICAMP), Bertrand Russell Avenue, Campinas, São Paulo 13083-865 Brazil; 2 Department of Food and Nutrition, School of Food Engineering, University of Campinas (UNICAMP), Campinas, São Paulo 13083-852 Brazil

**Keywords:** Prostate cancer, Jaboticaba, TRAMP, Epithelial-to-mesenchymal transition, Steroid hormones

## Abstract

Androgen deprivation therapy (ADT) is recommended for treating prostate cancer (PCa) that relapses after first-line approaches. However, tumors often evolve to a highly aggressive and metastatic castration-resistant phenotype (CRPC). Epithelial-to-mesenchymal transition (EMT) stands out due to its contribution to hormone resistance and metastatic spread. We investigated the potential of a jaboticaba peel extract (JPE) as an adjuvant agent in CRPC tumors from the Transgenic Adenocarcinoma of Mouse Prostate (TRAMP) model, focusing on EMT regulation. Sixteen-week-old mice underwent surgical and chemical castration (10 mg/Kg enzalutamide), alone or in combination with JPE (5.8 g/Kg). Control animals were sham-castrated and received either placebo or the plant extract. JPE reduced the ratio of widespread prostatic tumors in mice undergoing ADT, despite not affecting metastases. It also reduced the protein levels of the EMT drivers ZEB1 and N-Cadherin, apart from contributing to the maintenance of a periacinar layer primarily composed of smooth muscle cells. Mechanistically, JPE-induced effects in castrated mice involved decrease of AR protein expression as well as ERβ beneficial actions, which included a putative stimulation of TGF-β tumor-suppressive actions. In conclusion, JPE prevented the progression of poorly differentiated tumors with CRPC traits in the TRAMP model by hampering EMT and modulating steroid hormone and TGF-β signaling.

**Supplementary Information:**

The online version contains supplementary material available at 10.1007/s10735-026-10851-x.

## Introduction

Prostate cancer (PCa) is the most common in men in 112 countries and represents 15% of male malignancies. It has been estimated that the number of new diagnosed cases per year will rise from 1.4 million in 2020 to 2.9 million in 2040 (James et al. 2024). Androgen deprivation therapy (ADT) is the standard protocol for the treatment of tumors which relapse after radical prostatectomy and/or radiotherapy (Fang and Zhou 2019). Nevertheless, in 12–24 months, around 35% of the patients undergoing ADT show disease recurrence due to alternative mechanisms in androgenic signaling, which include adrenal or intratumoral production of these hormones, androgen receptor (AR) overexpression due to gene amplification or enhanced transcription, and also alternative splicing or mutations in the gene (Guerrero et al. 2013; Rice et al. 2019; Cheng et al. 2019; Cerasuolo et al. 2020; Wang et al. 2021). In these settings, PCa is referred to as castration-resistant prostate cancer (CRPC) and accounts for the majority of disease-related deaths (Rice et al. 2019; Cheng et al. 2019).

Enzalutamide is a non-steroidal AR antagonist which binds competitively and with high-affinity to this receptor, thereby preventing its nuclear translocation and leading to apoptosis induction and inhibition of proliferation in CRPC cells (Rice et al. 2019; Abazid et al. 2019; Nicola et al. 2021; Wang et al. 2021). For this reason, enzalutamide has been approved by the U.S. Food and Drug Administration (FDA) for the treatment of this advanced stage of PCa since 2012 (Avendaño López and Menendez 2015). Nevertheless, despite its initial clinical efficacy, tumor cells can acquire resistance to enzalutamide by both AR-mediated or independent mechanisms, which collectively favor CRPC progression to an incurable and lethal stage, since more than 90% of patients develop metastases (Rice et al. 2019; Cheng et al. 2019). At the same time, the direct and indirect involvement of estrogenic signaling in the development of CRPC has been documented, through mechanisms mediated by estrogen receptors alpha (ERα) and beta (ERβ) (Bonkhoff 2018; Qu et al. 2020). In particular, changes in the ERα/ERβ ratio in affected tissues are critical determinants for the variability in estrogen-dependent effects during cancer progression (Cipolletti et al. 2018).

Metastases development is directly related to the occurrence of epithelial-to-mesenchymal transition (EMT), a process of cell plasticity whereby cells lose their epithelial features, such as adhesion and apicobasal polarity, and acquire mesenchymal traits, including enhanced migratory and invasive capacity towards the surrounding stroma (Delgado-Bellido et al. 2017; Luo et al. 2020; Wei et al. 2021). During cancer-associated EMT, the expression of epithelial tight-junction proteins, including E-Cadherin and occludin, is downregulated in tumor cells, which simultaneously acquire mesenchymal markers, such as vimentin, N-Cadherin and alpha smooth muscle actin (αSMA) (Delgado-Bellido et al. 2017; Miao et al. 2017; Dongre and Weinberg 2019; Luo et al. 2020; Wei et al. 2021; Haerinck et al. 2023). Furthermore, it has been reported that EMT is stimulated in PCa when androgen responsiveness is lost, thus pointing to its involvement in the development of hormone-refractory disease, including upon enzalutamide treatment (Sun et al. 2012; Cai et al. 2020; Choi et al. 2021; Pang et al. 2021).

In vivo studies on PCa progression have been facilitated by animal models, such as the Transgenic Adenocarcinoma of Mouse Prostate (TRAMP), which mimics the progression of human disease and enables the control of environmental conditions able to interfere with its development (Greenberg et al. 1995; Balmain 2002; Berquin et al. 2005). In these mice, PCa development is driven by the expression of the simian virus 40 (SV40) Large T antigen under the control of rat probasin promoter in prostatic epithelial cells, where it acts as an oncoprotein that inactivates tumor-suppressor genes, such as pRB and p53 (Greenberg et al. 1995; Gelman 2016). Thus, beginning at 6 weeks of age, male TRAMP mice present nuclear changes within the prostate glands consistent with prostatic intraepithelial neoplasia (PIN), which later progress to well- and poorly differentiated adenocarcinomas (Gingrich et al. 1999; Kaplan-Lefko et al. 2003). Additionally, approximately 19% of the animals develop metastases between 18 and 24 weeks of age, primarily in the lungs and periaortic lymph nodes (Gingrich et al. 1996, 1999).

In this setting, taking into consideration that EMT is simultaneously involved in the development of metastases and castration resistance, novel adjuvant treatments for CRPC that target this mechanism are urgently needed. Collectively, these therapies should not only be able to disrupt essential pathways for tumorigenesis and progression but also maintain pharmacological safety, thereby improving both patient quality of life and overall survival. In this sense, several fruits have been under investigation aiming to prevent, or at least delay, the development of different tumor types due to the antiproliferative, antiangiogenic, antimetastatic and antioxidant properties of bioactive compounds found in their peels, seeds or leaves (Kris-Etherton et al. 2002; Reddy et al. 2003; Domeneghini and Lemes 2011; Wang et al. 2014; Kallifatidis et al. 2016; Costea et al. 2020). Among these, the Brazilian berry (*Myrciaria jaboticaba*), a native Brazilian species commonly known as "jaboticaba”, stands out due to the high anthocyanin content in its fruit peel, especially cyanidin-3-O-glucoside (approximately 80%) and delphinidin-3-O-glucoside (approximately 20%), which gives the fruit its typical deep purple to black color. Studies have shown that the antitumor effects of anthocyanins are largely attributed to their ability to inhibit cell proliferation, angiogenesis, invasion and metastases, while promoting cell cycle arrest, differentiation and apoptosis, as well as exerting anti-mutagenic, anti-inflammatory and antioxidant activities (Sorrenti et al. 2015; Lin et al. 2017). Additionally, other constituents, such as hydrolysable tannins, are also present in jaboticaba peel and contribute to its antioxidant potential (Leite-Legatti et al. 2012; Inada et al. 2021). However, although the antitumor effects of polyphenols have been classically attributed to their antioxidant properties, recent evidence indicates that their anticancer efficacy is especially associated with pro-oxidant and damage-inducing actions in tumor cells, apart from their effects on enzymes, intracellular transduction pathways and membrane and/or nuclear receptors, thereby suggesting the involvement of unknown mechanisms to be investigated (Amawi et al. 2017; Cipolletti et al. 2018).

In this sense, the jaboticaba peel extract (JPE) developed and patented by our research group has demonstrated promising effects, as it contains a wide range of bioactive compounds and enables the control over the ingested dose (Lamas et al. 2018). Furthermore, it is well established that administering these compounds as part of whole extracts offers advantages over ingesting them in isolated form, since their synergy lead to broader chemopreventive effects (Trombino et al. 2004; Milde et al. 2007; Inada et al. 2021). Finally, the absence of notable side effects, particularly in comparison to conventional chemotherapeutic agents, and the possibility to economically exploit a fruit fraction that lacks commercial interest, encourage further investigations into the chemopreventive potential of bioactive compounds from jaboticaba peel (Reddy et al. 2003; Kallifatidis et al. 2016; Wang et al. 2016; de Souza et al. 2025).

Based on the above, we have previously addressed the effects of JPE administration on the progression of in situ lesions in the dorsolateral prostate of TRAMP mice undergoing ADT and found that the beneficial effects of the extract are largely dependent on the androgenic reliance of the disease (Rezende et al. 2025). Nevertheless, given the frequent emergence of poorly differentiated tumors following castration in these mice (Kaplan-Lefko et al. 2003), as well as the remarkable histoarchitectural changes that support the development of such advanced lesions, this study aimed to further investigate the JPE effects on poorly differentiated CRPC in the TRAMP model, focusing on EMT modulation and metastatic development.

## Materials and methods

### Jaboticaba peel extract (JPE)

Fresh jaboticabas of the "Sabará” variety (*Myrciaria jaboticaba* (Vell.) O. Berg) were purchased in the Wholesale Supply Center (CEASA), Campinas, São Paulo, Brazil. Fruits were manually washed and peeled and the peels were subsequently grinded, lyophilized and frozen at − 20 °C until extract production. The method used for JPE production was developed and patented (BR 102017005462-4) by our research group, as described in Maróstica Júnior et al. (2017) and Lamas et al. (2018). Briefly, extract production consisted of dissolving lyophilized peel powder in ethanol, followed by solvent removal via rotary evaporation. Importantly, all of the extract used in this study was produced immediately before usage from a single batch of raw material obtained from the same supplier, thereby ensuring controlled farming and environmental conditions. The detailed characterization and quantification of the bioactive compounds present in the JPE obtained through this extraction method, the assessment of its in vitro antioxidant activity, as well as the definition of the in vivo administration dose were previously carried out and published by our group (Lamas et al. 2018). The main constituents of the freeze-dried extract, as determined by Ultra-Performance Liquid Chromatography Coupled with Electrospray Ionisation/Quadrupole-Time-of-Flight-Mass Spectrometry (UPLC-ESI-QTOF-MS/MS), were 13.28 mg/g cyanidin-3-O-glucoside (C3G), 1.428 mg/g delphinidin-3-O-glucoside (D3G), 0.196 mg/g ellagic acid, 0.02 mg/g rutin and 0.017 mg/g gallic acid (Lamas et al. 2018). Other bioactive compounds were also detected, including: HHDP-galloylglucose, bis-HHDP-glucose (casuarin), bis-HHDP-glucose isomer (pedunculagin), HHDP- galloylglucose isomer, (-)-epicatechin, galloyl-bis-HHDP- glucose (casuarinin), galloyl-bis-HHDP-glucose (casuarictin), HHDP-digalloylglucose (tellimagrandin I), kaempferol hexoside, chlorogenic acid, HHDP-trigalloylglucose (tellimagrandin II), pentagalloyl hexose, myricetin-rhamnoside, quercetin-3-rhamnoside (quercitrin), quercetin and narigenin (Lamas et al. 2018).

### Animals and experimental procedure

**Fig. 1:**
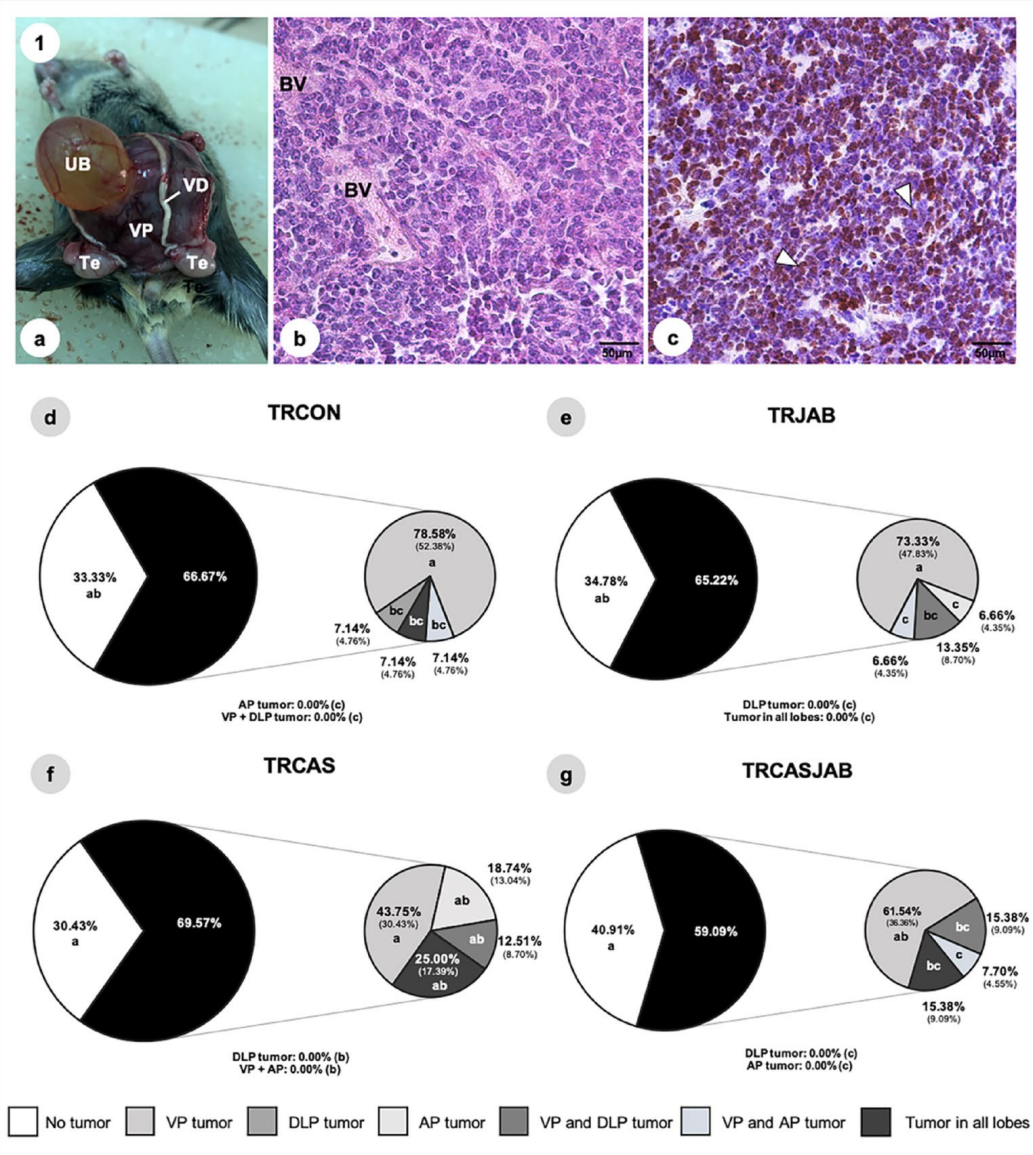
Evaluation of macroscopic and poorly differentiated tumors in TRAMP mice from the experimental groups. **A** Gross aspect of a palpable tumor encompassing the ventral prostate. **B** Representative photomicrograph depicting tumor morphology under light microscopy. Hematoxylin and eosin (H&E) staining. **C** SV40 Large T immunostaining of a tumor section showing diffuse antigen reactivity and confirming the prostatic origin of the observed tumors. **D**–**G** Tumor frequencies in the prostatic lobes at euthanasia. The pie chart on the left of each panel shows the percentage of mice with (black area) or without (white area) tumors. The smaller pie chart on the right shows the incidence of poorly differentiated tumors in the prostatic lobes, as indicated in the legend. Values are expressed as percentages of the total number of mice (numbers in parentheses) or relative to the amount of tumor-bearing animals in each group (bold numbers). Different lowercase letters indicate statistically significant differences between two categories of tumor distribution within a specific experimental group (P < 0.05). For detailed information about such differences, refer to Table 1. Abbreviations: *AP:* anterior prostate; *BV*: blood vessel; *DLP:* dorsolateral prostate; *UB:* urinary bladder; *Te:* testis; *VD:* vas deferens; *VP:* ventral prostate

A total of eighty-nine male TRAMP mice (C57BL/6-Tg(TRAMP)8247Ng/J X FVB/NJ)F1/J) were used in this study. The animals were supplied by the Multidisciplinary Center for Biological Research in Laboratory Animal Science (CEMIB) at the University of Campinas (UNICAMP) and were kept in the animal facility of the Structural and Functional Biology Department (Anatomy division) at the Institute of Biology under pathogen-free and stable conditions of temperature (20-26ºC), humidity (40–60%) and luminosity (12h light/dark cycle). After the acclimatization period, and upon reaching 16 weeks of age, the mice were randomly assigned to four experimental groups. Two groups underwent sham castration and, after 48h, received placebo treatment by gavage with the dilution vehicles for JPE and enzalutamide (*TRAMP Control Group* - *TRCON*; n = 21) or 5.8 g/Kg of JPE diluted in tap water (*TRAMP Jaboticaba Group* - *TRJAB*; n = 23) (Lamas et al. 2018). Considering this dose, each experimental animal received the following daily amounts of the main JPE compounds: 9.3 mg of cyanidin-3-O-glucoside, 1 mg of delphinidin-3-O-glucoside, 0.14 mg of ellagic acid, 0.014 mg of rutin and 0.012 mg of gallic acid (Lamas et al. 2018). The remaining groups underwent orchiectomy and, after 48h, were chemically castrated through oral gavage with 10 mg/kg of enzalutamide (MDV3100) (*RayBiotech*, Peachtree Corners, USA) diluted in a vehicle containing 1% carboxymethylcellulose (CMC) (*Labsynth*, Diadema, Brazil), 1% Tween 80 (*Labsynth*, Diadema, Brazil) and 5% dimethyl sulfoxide (DMSO) (*Sigma-Aldrich*, St. Louis, USA) (Guerrero et al. 2013; Nicola et al. 2021). This ADT approach was applied either alone (*TRAMP Castrated Group* - *TRCAS*; n = 23) or in combination with 5.8 g/Kg of JPE (*TRAMP Castrated* + *Jaboticaba Group* - *TRCASJAB*; n = 22). All animals were treated five times per week and had free access to tap water and the same solid diet (*Nuvilab, Quimtia*, Colombo, Brazil) ad libitum. At the end of 6 weeks of treatment, mice were weighed in a semianalytic scale AS 5500 (*Marte Científica*, São Paulo, Brazil), anesthetized with 2% xylazine chlorhydrate (5 mg/Kg i.m.) (*König*, Mairinque, Brazil) and 10% ketamine chlorhydrate (60 mg/Kg i.m.) (*Ceva Saúde Animal*, Paulínia, Brazil) and euthanized. During the procedure, we harvested periaortic lymph nodes, lungs and prostate samples from individual lobes or poorly differentiated tumors. The latter were initially identified at euthanasia based on their gross aspect in contrast to the coiled tubular structures typical of normal prostatic lobes (Fig. 1A). Lesion grading was subsequently confirmed using previously established histopathological criteria (Gingrich et al. 1999; Kaplan-Lefko et al. 2003; Berman-Booty et al. 2011). The proportion of tumor-free mice was statistically analyzed among the experimental groups (Fig. 1). Prostatic lobes harvested from these animals were assigned to other studies conducted by our research group. The experimental protocol of this study was approved by the Committee for Ethics in Animal Research of the University of Campinas (protocol nº 6266–1/2023). Experimental sample size was calculated with the GraphPad StatMate 2 software (available for download at: www.graphpad.com) using the following parameters: standard deviation = 0.25 (based on former studies in the literature, on the minimal difference to be detected among experimental groups = 0.65); significance level = 0.05 and statistical test power = 95%. During all stages of the experiment, authors (F.R.S. and F.M.) were aware of the group allocation of each mouse.

**Table 1:** Distribution of the incidences of poorly differentiated tumors in the prostatic lobes from TRAMP mice of the experimental groups

Tumor distribution	Experimental groups			
	TRCON (n = 21)	TRJAB (n = 23)	TRCAS (n = 23)	TRCASJAB (n = 22)
No tumor	33.33%^ab^	34.78%^ab^	30.43%^a^	40.91%^a^
VP tumor	52.38%^a^	47.83%^a^	30.43%^a^	36.36%^ab^
DLP tumor	4.76%^bc^	0.00%^c^	0.00%^b^	0.00%^c^
AP tumor	0.00%^c^	4.35%^c^	13.04%^ab^	0.00%^c^
VP and DLP tumor	0.00%^c^	8.70%^bc^	8.70%^ab^	9.09%^bc^
VP and AP tumor	4.76%^bc^	4.35%^c^	0.00%^b^	4.55%^c^
Tumor in all lobes	4.76%^bc^	0.00%^c^	17.39%^ab^	9.09%^bc^

Different lowercase letters indicate statistically significant differences within the groups (P < 0.05)

Statistical test: Two-way ANOVA (Tukey`s post hoc test)

### Histochemistry and immunohistochemistry

Tissue samples were submitted to immunostaining protocols to localize the proteins of interest in the various organs analyzed. The antigens E-Cadherin, αSMA, vimentin and N-Cadherin were detected in poorly differentiated tumor samples, which were also submitted to Hematoxylin–Eosin staining to provide information about their morphology. Additionally, periaortic lymph nodes and lungs were stained for SV40 Large T antigen. All tissue sections were obtained with 5 µm thickness in a Hyrax M60 microtome (*Zeiss*, Oberkochen, Germany) and, when used for immunohistochemistry, were collected in silanized slides. Antigen retrieval was performed by incubating the sections in a 10 mM citrate buffer (pH 6.0) on a microwave oven. Endogenous peroxidase blockage was achieved with incubation in 0.3% H_2_O_2_ in methanol for 20 min, whereas nonspecific protein interactions were blocked through incubation in 1–3% bovine serum albumin (BSA) diluted in Tris-buffer saline + Tween 20 (TBS-T) solution for 1h at room temperature. For antigen detection, sections were incubated overnight at 4 °C with some of the primary antibodies listed in Supplementary Table S1, diluted 1:100–1:1500 in TBS-T buffer or in 1% BSA, according to the specific characteristics of each antigen. After a series of washes, sections were incubated with the HRP-conjugated secondary antibodies goat anti-rabbit IgG (W4011) and goat anti-mouse IgG (W4021) (*Promega Corporation*, Madison, USA), and antigen reactivity was developed using a diaminobenzidine (DAB) chromogenic solution (*Sigma-Aldrich*, St. Louis, USA). Sections were then counterstained with Harris Hematoxylin, dehydrated, mounted in *Entellan™* (Merck*, Darmstadt*, Germany) and photographed in the microscope *Nikon Eclipse E-400* (*Nikon*, Tokyo, Japan). The reactivities of E-Cadherin, αSMA and vimentin were quantitatively assessed in poorly differentiated prostatic tumors by analyzing ten randomly selected fields per animal at 400× magnification. Specifically, for E-Cadherin, quantification was performed by measuring the area of positive immunolabeling using the software *ImageJ* (*Image Analysis and Processing in Java*). In contrast, for vimentin and αSMA, quantitative evaluation of reactivity was performed using a grid system with 864 intersection points applied to the microscopic fields (Weibel 1963). In each field, the number of intersections overlapping areas positive for the specific antigen was counted and expressed as a percentage of the total points.

### Metastases foci analysis

Samples of lungs and periaortic lymph nodes were harvested from randomly selected animals in each experimental group and analyzed for the presence of metastases using SV40 Large T antigen immunolabeling, as described in the previous section. The prostatic origin of the metastatic lesions was confirmed by identifying cells positive for this antigen, following the procedure described by Gingrich et al. (1996) and (Copeland et al. 2013). Briefly, paraffin-embedded tissues were sectioned at 5µM-thickness at regular intervals, and at least three sections from each sample were harvested for immunohistochemistry. A mouse was considered positive for metastasis when at least one metastatic lesion was detected by immunohistochemistry in its lymph node or lung samples.

### Western blotting

Samples from poorly differentiated tumors in each experimental group were frozen in liquid nitrogen and lysed on ice using protein extraction buffer (150 mM NaCl, 50mM Tris–HCl, 1% Triton, 0,1% SDS, pH = 8.0) supplemented with 1% aprotinin from bovine lung (*Sigma-Aldrich*, St. Louis, USA). Protein extraction, determination of total protein concentration and Western Blotting were performed as previously described by Kido et al. (2020) (Supplementary Table S1). The secondary antibodies used were goat anti-rabbit IgG (W4011) e goat anti-mouse IgG (W4021) (*Promega Corporation*, Madison, USA). Protein band detection was carried out by incubating the membranes in a chemiluminescent solution (*Thermo Fisher Scientific*, Waltham, USA), followed by fluorescence capture using the Gene Gnome detection system and *GeneSnap* image acquisition software (*Syngene,* Frederick, USA). The endogenous control for the experiments was obtained by determining the total protein content in each sample through membrane staining with Ponceau S (*Sigma-Aldrich*, Saint Louis, USA), as described by Romero-Calvo et al. (2010) and Rezende et al. (2025). Briefly, Ponceau S stains all proteins non-specifically within each lane of the blot, enabling the use of a wide range of molecular weights (approximately 90%) instead of a single protein for densitometric analysis and quantification (Supplementary Fig. S1). The intensity of antigen bands in the different experimental groups was quantified by densitometry using the software Image J (Image Analysis and Processing in Java) and the results are presented as mean optical densities relative to the TRCON group, which was set as 100.0%. Representative Western Blotting membranes used for determination of protein levels for each antigen are provided in the Supplementary Material.

### Statistical analyses

Parameters quantified in the poorly differentiated tumor and metastases incidence evaluations, as well as in the immunohistochemical and Western Blotting experiments were analyzed using the GraphPad Prism 8.4.0® for MacOS (*GraphPad Software*, San Diego, USA). One-way analysis of variance (ANOVA), followed by Tukey’s post hoc test, was used to compare means of parametric data. Values with non-Gaussian distribution were analyzed using Kruskal–Wallis test followed by Dunn’s post hoc test. Two-way ANOVA with Tukey’s post hoc was used to evaluate tumor incidence across the different prostatic lobes in the experimental groups. All analyses were performed at 5% significance level and results were expressed as mean ± standard error of mean (SEM) (Montgomery 1991; Zar 1999).

## Results

### JPE administration counteracted the aggressiveness of poorly differentiated prostatic tumors in TRAMP mice under hormonal ablation

The incidence and growth rate of poorly differentiated prostatic tumors was evaluated in the experimental groups. For this purpose, only mice free of palpable tumors at 16 weeks old were included in the study, in such a manner that the presence or absence of tumors at the end of 22 weeks could be solely attributed to the different experimental conditions in each group. The growth rate was extrapolated from tumor weight and its ratio in relation to body weight at the euthanasia. Additionally, these tumors often become macroscopic and palpable and may comprise one or all prostatic lobes, thus providing information about their aggressiveness (Fig. 1A). Microscopically, they are characterized by uncontrolled proliferation of epithelial cells, depicting remarkable disorganization of prostatic morphology, with the glands being replaced by cell sheets containing nuclear and cytoplasmic atypias (Fig. 1B) Importantly, the prostatic origin of such tumors was confirmed by the immunolabeling of SV40 Large T antigen, which is specifically expressed in the prostatic epithelium of the TRAMP model due to the fusion between its gene and probasin promoter region (Fig. 1C).

No differences were registered considering tumor or tumor/body weight ratio in the experimental groups. Nevertheless, final body weight was significantly reduced in the castrated groups (TRCAS and TRCASJAB) as compared to their respective control groups (P < 0.05 in relation to TRCON and P < 0.01 in relation to TRJAB, respectively) (Supplementary Fig. S2; Supplementary Table S2). Additionally, we verified that all the experimental groups registered similar rates of tumor-free mice, thus showing that JPE did not affect this parameter in any of the androgenic reliance scenarios tested (Fig. 1D–G; Table 1). Hormonal ablation, in turn, had a striking influence on tumor aggressiveness since these malignant lesions, which were predominantly restricted to the ventral prostate in sham-castrated mice, equally spread to two or all the prostatic lobes following surgical and chemical castration (Fig. 1F–G; Table 1). Nevertheless, in this setting, we found that JPE was able to increase the rate of tumor-free mice within the TRCASJAB group, statistically overcoming the proportion of individuals whose tumors disseminated beyond a specific lobe (Fig. 1F and G; Table 1). This unique finding, which was not registered in the TRCAS group, suggests that JPE may have displayed chemopreventive actions under androgen ablation, restricting tumor aggressiveness.

**Fig. 2:**
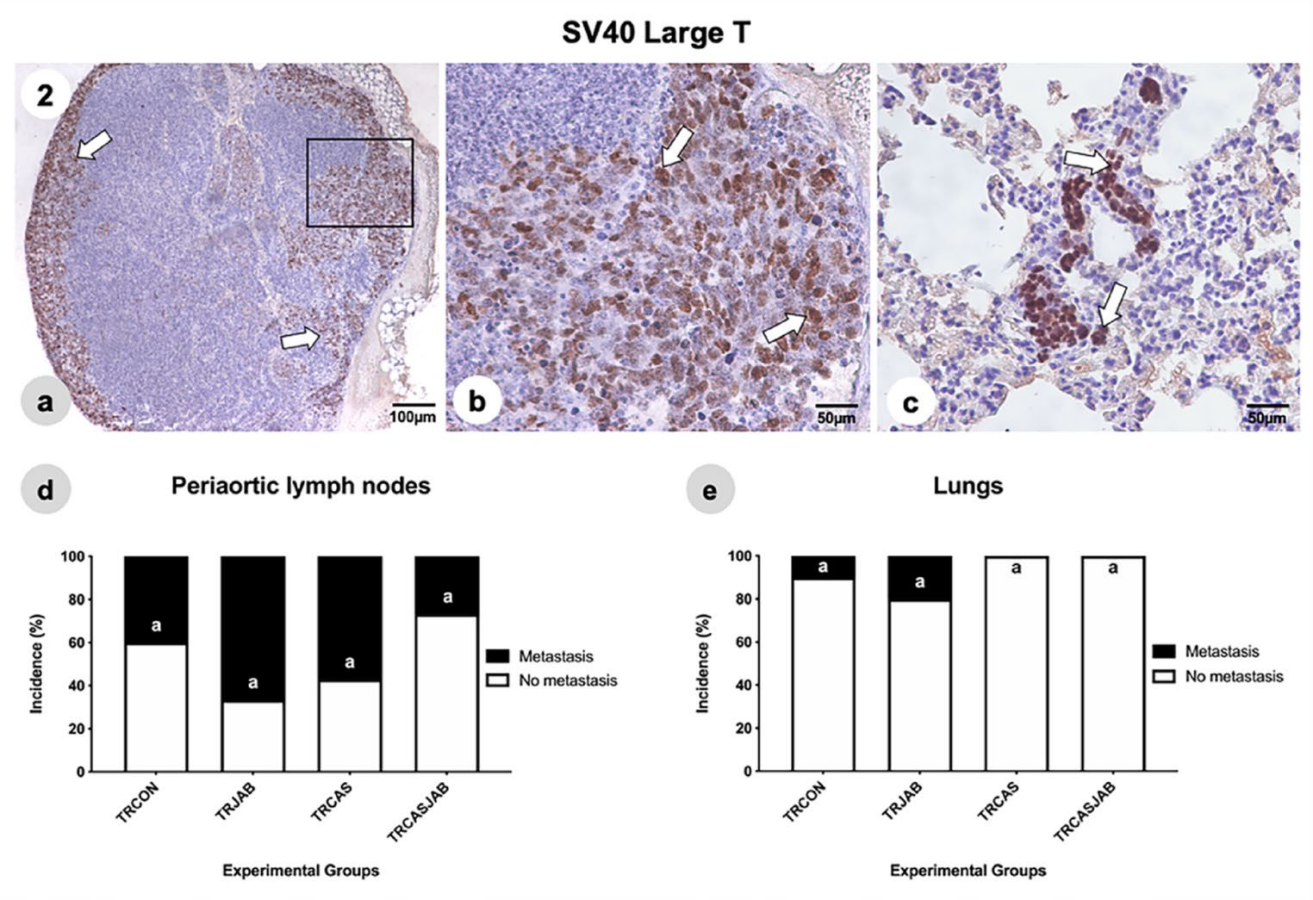
Effects of the different experimental conditions on the incidence of metastases in periaortic lymph nodes (**A**–**B**) and lungs (**C**) from TRAMP mice. Prostatic origin of metastatic tumor cells was confirmed by SV40 Large T antigen immunolabeling (white arrows). Panel **B** is a higher magnification of the area inside the rectangle in panel A. **D**–**E** Graphs with the incidences of lymphatic and pulmonary metastases in the experimental groups. Similar lowercase letters indicate no statistical differences (P > 0.05)

We also investigated the incidence of metastasis in periaortic lymph nodes and lungs of TRAMP mice from the experimental groups by immunolabeling SV40 Large T antigen in these organs (Fig. 2A–C). Despite not having detected pulmonary metastatic lesions in TRCAS and TRCASJAB groups, our results did not show any statistically significant differences (P > 0.05) among the tissue samples analyzed (Fig. 2D and E; Table 2).

**Table 2:** Incidences of metastatic lesions in TRAMP mice from the experimental groups. Numbers in parentheses indicate the number of animals with metastasis in a specific organ over the total number of samples analyzed

Organs	Experimental groups			
	TRCON	TRJAB	TRCAS	TRCASJAB
Periaortic lymph node	40.00%^a^ (6/15)	66.67%^a^ (10/15)	57.14%^a^ (8/14)	26.67%^a^ (4/15)
Lung	10.00%^a^ (1/10)	20.00%^a^ (2/10)	0.00%^a^ (0/10)	0.00%^a^ (0/10)

Similar lowercase letters indicate no statistical differences among the groups (P < 0.05)

Statistical test: Two-way ANOVA (Tukey`s post hoc test)

In subsequent analyses, only poorly differentiated prostate tumors with confirmed histopathological grading were included.

### ADT favors estrogenic signaling and the development of tumors with CRPC traits in TRAMP mice but JPE association circumvents this harmful milieu by promoting ERβ actions

**Fig. 3:**
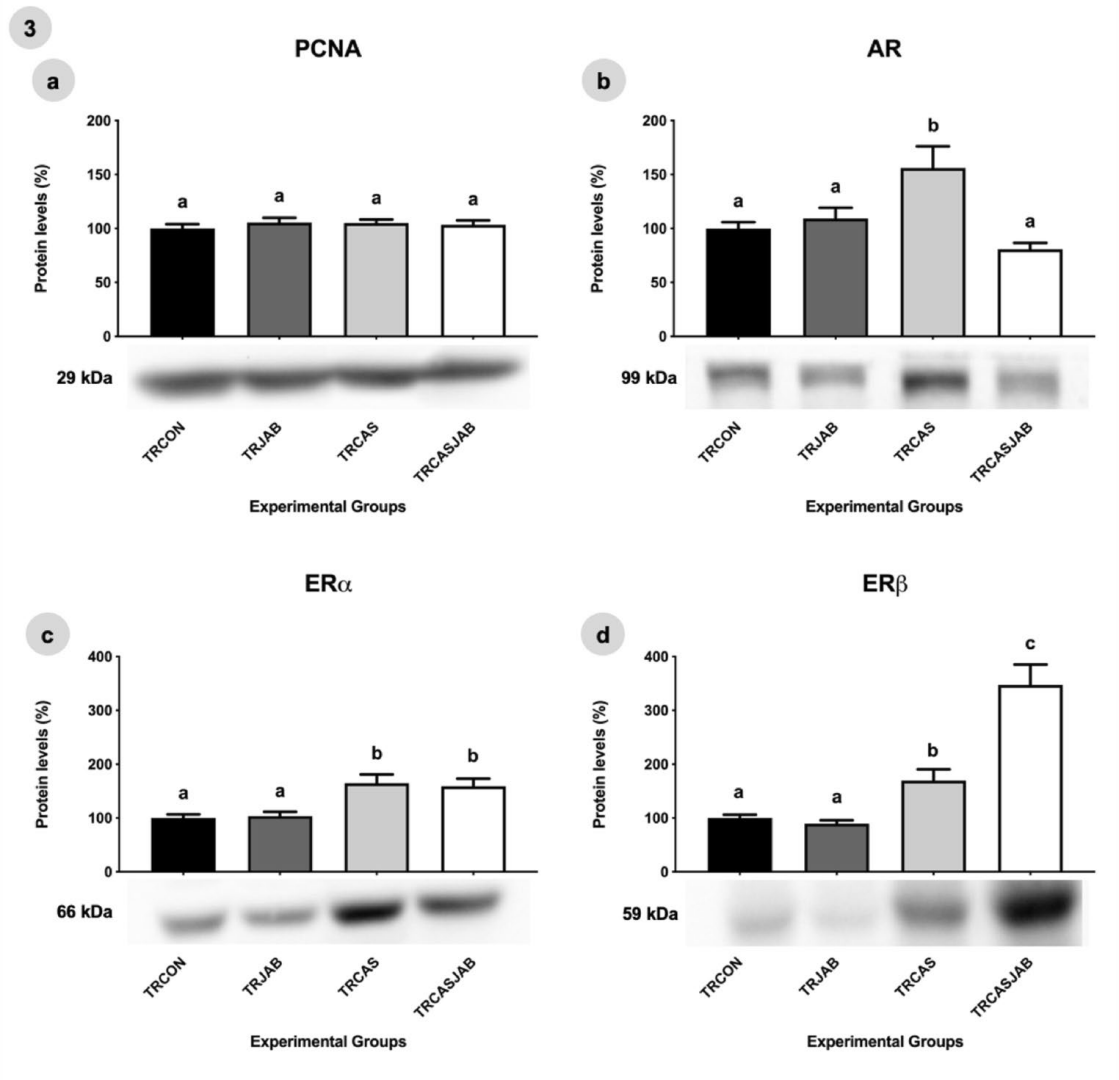
Protein expression of proliferative and steroid hormone signaling markers in the experimental groups. **A**–**D** Protein levels of the proliferating cell nuclear antigen (PCNA) (**A**), androgen receptor (AR) (**B**), estrogen receptor alpha (ERα) (**C**) and estrogen receptor beta (ERβ) (**D**) as determined by Western Blotting in poorly differentiated tumors. Values are expressed as mean ± standard error of mean in relation to the protein levels of the TRCON group. For all analyses, different lowercase letters indicate statistically significant differences in the comparison between two experimental groups (P < 0.05)

Considering the essential role of steroid hormone signaling in regulating tumor cell proliferation during CaP progression, we evaluated the protein levels of PCNA, a proliferative marker, as well as androgen and estrogen receptors in poorly differentiated tumors from TRAMP mice treated with JPE under hormone-naïve or androgen-ablation settings. Regarding PCNA, no statistically significant differences were registered among experimental groups (P > 0.05) (Fig. 3A; Table 3). On the other hand, we found a significant raise on AR levels following castration (156.1% in the TRCAS group vs. 100.0% in the TRCON group; P < 0.01), whereas JPE administration in this scenario was able to reverse this increase in AR protein expression (80.74% in the TRCASJAB; P < 0.001 in relation to TRCAS), reaching levels similar to those found in the TRCON group (Fig. 3B; Table 3). Collectively, these findings support the CRPC phenotype of the tumors observed upon castration, especially in the TRCAS group, in which the increased levels of AR in an androgen-ablated microenvironment may indicate ligand-independent actions of the receptor. Importantly, JPE displayed the ability to reverse this harmful scenario, reducing the levels of receptor expression.

**Table 3:** Protein levels of the different antigens as determined by Western Blotting in poorly differentiated tumors from TRAMP mice of the experimental groups. Values are expressed as mean ± standard error of mean in relation to the protein levels of the TRCON group

Antigen	Experimental groups			
	TRCON	TRJAB	TRCAS	TRCASJAB
PCNA [29 kDa]*	100.0 ± 3.97^a^	105.5 ± 4.33^a^	105.0 ± 3.36^a^	103.5 ± 4.04^a^
AR [99 kDa]*	100.0 ± 5.82^a^	109.3 ± 9.87^a^	156.1 ± 19.94^b^	80.74 ± 6.03^a^
ERα [66 kDa]**	100.0 ± 7.09^a^	103.6 ± 8.05^a^	164.8 ± 16.30^b^	159.3 ± 13.82^b^
ERβ [59 kDa]*	100.0 ± 6.34^a^	89.90 ± 5.87^a^	169.6 ± 20.72^b^	347.3 ± 38.08^c^
E-Cadherin [135 kDa]**	100.0 ± 10.49^a^	91.79 ± 12.45^a^	73.61 ± 9.02^a^	70.79 ± 7.08^a^
αSMA [42 kDa]**	100.0 ± 7.64^a^	134.9 ± 11.57^ab^	136.9 ± 15.07^ab^	173.0 ± 20.20^b^
Vimentin [54 kDa]**	100.0 ± 13.21^a^	67.33 ± 9.12^a^	51.92 ± 6.32^a^	78.26 ± 8.20^a^
N-Cadherin [140 kDa]**	100.0 ± 6.55^ac^	132.8 ± 9.29^ab^	185.3 ± 20.29^b^	89.55 ± 4.03^c^
ZEB1 [200 kDa]*	100.0 ± 9.56^a^	53.64 ± 4.60^a^	187.3 ± 45.85^b^	101.5 ± 11.91^a^
Snail [29 kDa]*	100.0 ± 4.56^a^	100.7 ± 4.88^a^	115.2 ± 9.62^a^	97.69 ± 10.34^a^
Latent TGF-β1 [52 kDa]**	100.0 ± 4.78^ab^	77.13 ± 5.26^a^	138.9 ± 17.25^bc^	247.9 ± 38.31^c^
Mature TGF-β1 [25 kDa]*	100.0 ± 5.61^a^	83.94 ± 8.38^a^	170.6 ± 24.60^b^	238.8 ± 23.21^c^
Latent TGF-β2 [48 kDa]*	100.0 ± 3.79^ab^	78.92 ± 6.65^a^	142.1 ± 14.51^b^	217.7 ± 25.28^c^
Mature TGF-β2 [25 kDa]*	100.0 ± 4.52^a^	91.01 ± 8.60^a^	170.7 ± 20.45^b^	274.7 ± 31.85^c^
TGFβ-RI [55 kDa]*	100.0 ± 3.62^a^	81.42 ± 8.72^a^	136.6 ± 18.03^a^	268.5 ± 38.62^b^
TGFβ-RII [65 kDa]*	100.0 ± 4.80^a^	83.62 ± 4.49^ab^	75.92 ± 3.79^b^	96.13 ± 9.66^ab^

Different lowercase letters indicate statistically significant differences among the groups (P < 0.05)

Statistical tests: *One-way ANOVA (Tukey`s post hoc test) / **Kruskal–Wallis (Dunn’s post hoc test)

Regarding estrogenic signaling, no changes were registered in ERα and ERβ protein levels following isolated JPE treatment (P > 0.05). Nevertheless, both receptors demonstrated raised expression after ADT per se (164.8% and 169.6% of the TRCON group levels for ERα and ERβ, resulting in P < 0.01 and P < 0.05, respectively) (Fig. 3C and D; Table 3). On the other hand, while ERα showed similar protein levels upon JPE association in the ADT scenario (159.3% in the TRCASJAB group; P > 0.05), ERβ characterized an even higher enhancement of its expression, with significantly different values in relation to the TRCAS group (347.3% in the TRCASJAB; P < 0.0001) (Fig. 3C and D; Table 3). Overall, these data point to enhanced estrogenic signaling in the microenvironment of TRAMP mice poorly differentiated tumors following ADT and also suggest that, upon JPE association, this signaling is being mediated primarily by ERβ, considering the higher ratio between this receptor and ERα.

### JPE contributes to glandular architecture maintenance and counteracts EMT in poorly differentiated tumors from TRAMP mice undergoing ADT

Repression of E-Cadherin expression, primarily mediated by ZEB1 and Snail transcription factors, has been reported as a sufficient condition for EMT induction based on the essential role of this adhesion molecule in maintaining cell junctions and, hence, the epithelial phenotype (Delgado-Bellido et al. 2017; Nakazawa and Kyprianou 2017; Dicken et al. 2019; Luo et al. 2020; Wei et al. 2021). Conversely, acquisition of the mesenchymal phenotype has been associated with increased levels of vimentin, α-smooth muscle actin (αSMA) and N-Cadherin, among other proteins (Delgado-Bellido et al. 2017; Miao et al. 2017; Luo et al. 2020; Wei et al. 2021). Considering this background, we evaluated protein expression and tissue localization of these markers in poorly differentiated tumors of the experimental groups using Western Blotting and immunohistochemistry, respectively.

**Fig. 4:**
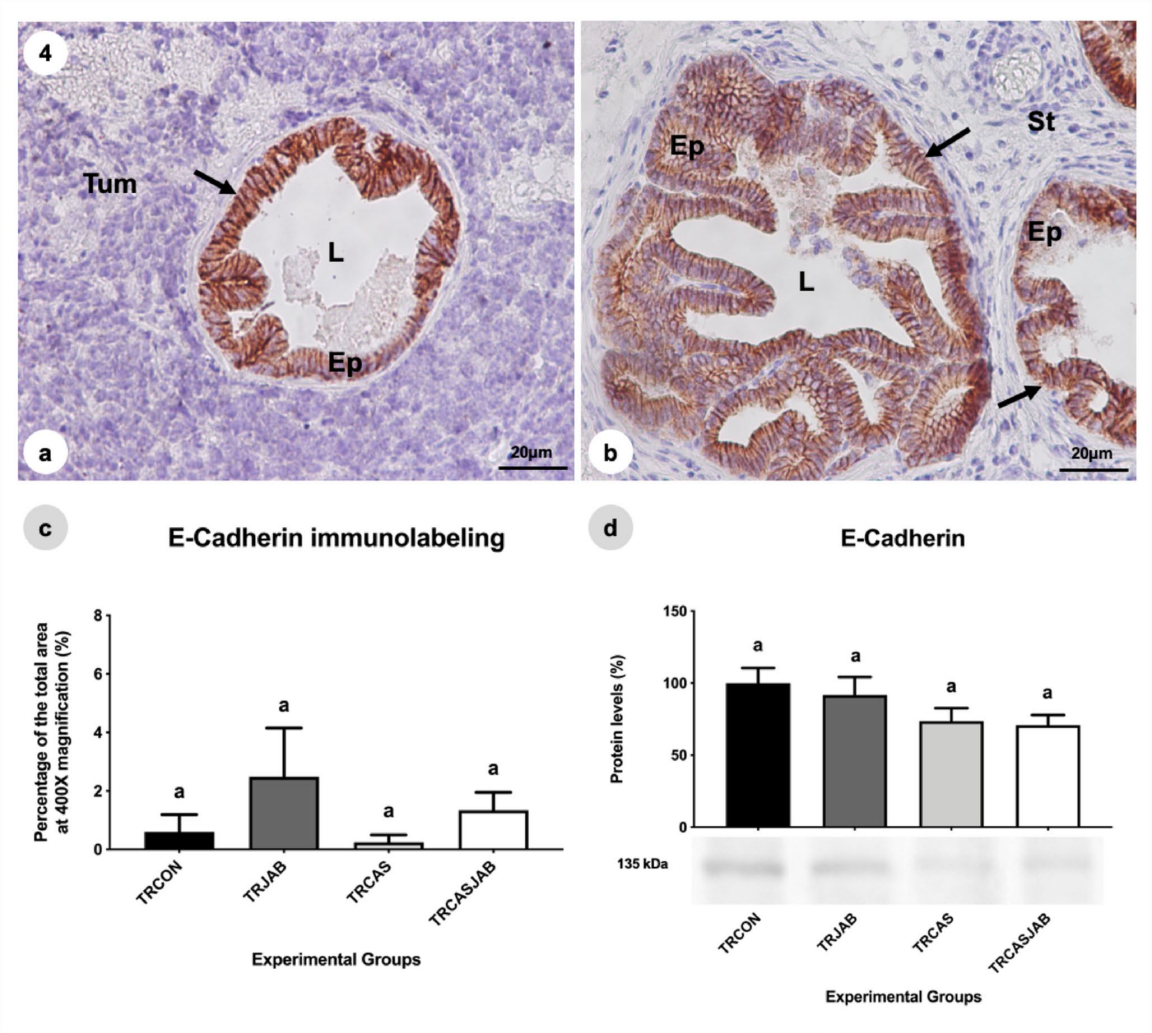
Analysis of E-Cadherin expression in poorly differentiated tumors from the experimental groups. **A**–**B** Representative photomicrographs of E-Cadherin immunostaining in poorly differentiated tumors (**A**) and in samples of the ventral prostate showing preserved glandular morphology (not included in this study and used as a reference) (**B**). Areas with positive immunolabeling are depicted by arrows. **C** Average percentages of the total microscopic field area showing E-Cadherin positivity in the tumors. **D** Protein levels of E-Cadherin in the different groups as determined by Western Blotting. Values are expressed as mean ± standard error of mean in relation to the protein levels of the TRCON group. Similar lowercase letters indicate no statistical differences (P > 0.05). Abbreviations: Ep: epithelium; L: lumen; St: stroma; Tum: tumor cells

Results showed that E-Cadherin staining followed a focal distribution that was limited to the remaining glands intermingled by poorly differentiated tumor cells, which did not present any reactivity for this antigen (Fig. 4A). Interestingly, at the same time, we also investigated E-Cadherin immunolabeling in tumor-free ventral prostate samples from our experimental groups and found a diffuse epithelial distribution for this molecule, regardless of the occurrence of proliferative lesions or healthy aspect of the prostatic epithelium (Fig. 4B). Based on these findings, we proceeded to the quantification of E-Cadherin positive areas in poorly differentiated tumors aiming to detect possible fluctuations in epithelial phenotype maintenance among experimental groups. On the contrary to our expectations, no significant differences were registered (P > 0.05) either for this parameter or for total protein levels of E-Cadherin (Fig. 4C and D; Tables 3 and 4).

**Table 4:** Relative areas showing E-Cadherin positivity in poorly differentiated tumors from the experimental groups. Values are presented as mean percentages of the total microscopic field area at 400X magnification ± standard error of the mean

Antigen	Experimental groups			
	TRCON	TRJAB	TRCAS	TRCASJAB
E-Cadherin	0.596 ± 0.596^a^	2.49 ± 1.67^a^	0.250 ± 0.250^a^	1.34 ± 0.612^a^

Similar lowercase letters indicate no statistical differences among the groups (P < 0.05)

Statistical test: Kruskal–Wallis (Dunn’s post hoc test)

**Fig. 5:**
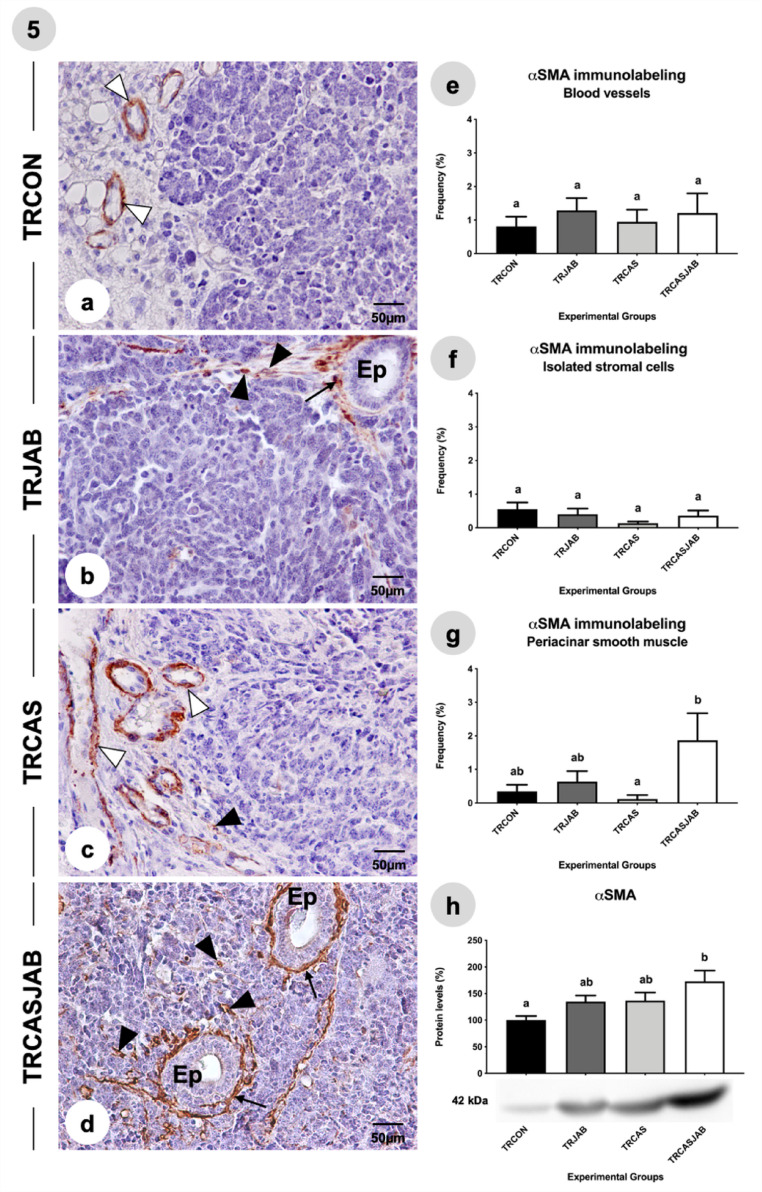
Analysis of smooth muscle alpha actin (αSMA) expression in poorly differentiated tumors from the experimental groups. **A**–**D** Representative photomicrographs of αSMA immunostaining. The antigen reactivity was counted according to the following categories: blood vessels (white arrowheads), isolated stromal cells (black arrowheads) and periacinar smooth muscle layer (black arrows). **E**–**G** Frequency distribution for each reactivity category. **H** Protein levels of αSMA in the different groups as determined by Western Blotting. Values are expressed as mean ± standard error of mean in relation to the protein levels of the TRCON group. For all analyses, different lowercase letters indicate statistically significant differences in the comparison between two experimental groups (P < 0.05). Abbreviations: Ep: epithelium

**Fig. 6:**
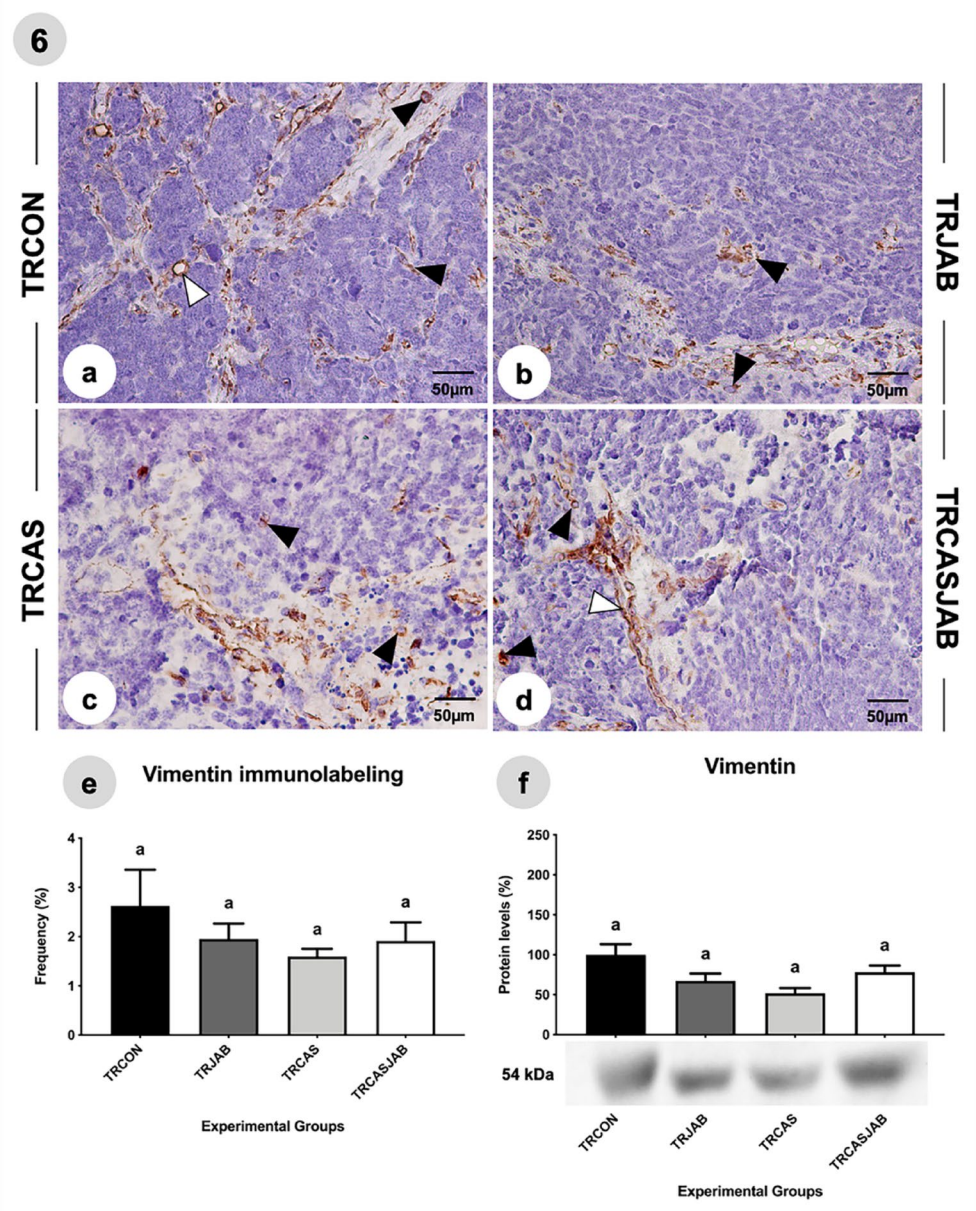
Analysis of vimentin expression in poorly differentiated tumors from the experimental groups. **A**–**D** Representative photomicrographs of vimentin immunostaining. Antigen reactivity in isolated stromal cells and blood vessels is indicated by black and white arrowheads, respectively. **E** Frequency distribution of vimentin immunolabeling. **F** Protein levels of vimentin in the different groups as determined by Western Blotting. Values are expressed as mean ± standard error of mean in relation to the protein levels of the TRCON group. Similar lowercase letters indicate no statistical differences (P > 0.05)

Considering αSMA, its staining was detected in three distinct tissue locations, namely: 1) blood vessels; 2) isolated stromal cells; and 3) periacinar smooth muscle layer. Therefore, the immunolabeling for this antigen was evaluated according to the same categories (Fig. 5; Table 5). Results did not show any statistically significant differences (P > 0.05) among the experimental groups regarding αSMA reactivity in blood vessels and isolated stromal cells (Fig. 5A–D, E and F; Table 5). On the other hand, the TRCASJAB group displayed significant raise in periacinar αSMA positivity when compared to the TRCAS group (1.87% vs. 0.12%, respectively; P < 0.05), whereas no other statistical differences were registered among the experimental groups (Fig. 5A–D and 5G; Table 5). Interestingly, total protein levels of αSMA progressively increased after castration (in the TRCAS group) and upon its association with JPE treatment (in the TRCASJAB group), thus yielding a significant difference between this latter group and the TRCON one (173.0% vs. 100.0%, respectively; P < 0.05) (Fig. 5H; Table 3). At the same time, as for vimentin reactivity and total protein levels, no significant differences were registered among the experimental groups (Fig. 6A–G; Tables 3 and 6).

**Table 5:** Smooth muscle alpha actin (αSMA) reactivity frequencies in poorly differentiated tumors from the experimental groups. Antigen positivity was counted according to the following categories: blood vessels, isolated stromal cells and periacinar smooth muscle layer. Values are expressed as mean percentages ± standard error of the mean

αSMA reactivity categories	Experimental groups			
	TRCON	TRJAB	TRCAS	TRCASJAB
Blood vessels*	0.137 ± 0.023^a^	0.298 ± 0.156^a^	0.200 ± 0.119^a^	0.393 ± 0.155^a^
Isolated stromal cells*	0.552 ± 0.199^a^	0.398 ± 0.172^a^	0.136 ± 0.047^a^	0.354 ± 0.162^a^
Periacinar smooth muscle**	0.346 ± 0.196^ab^	0.637 ± 0.313^ab^	0.118 ± 0.118^a^	1.87 ± 0.808^b^

Different lowercase letters indicate statistically significant differences among the groups (P < 0.05)

Statistical tests: *Kruskal–Wallis (Dunn’s post hoc test) / **One-way ANOVA (Tukey`s post hoc test)

**Table 6:** Vimentin reactivity frequencies in poorly differentiated tumors from the experimental groups. Values are expressed as mean percentages ± standard error of the mean

Antigen	Experimental groups			
	TRCON	TRJAB	TRCAS	TRCASJAB
Vimentin	2.62 ± 0.738^a^	1.95 ± 0.311^a^	1.60 ± 0.157^a^	1.91 ± 0.377^a^

Similar lowercase letters indicate no statistical differences among the groups (P < 0.05)

Statistical test: One-way ANOVA (Tukey`s post hoc test)

**Fig. 7:**
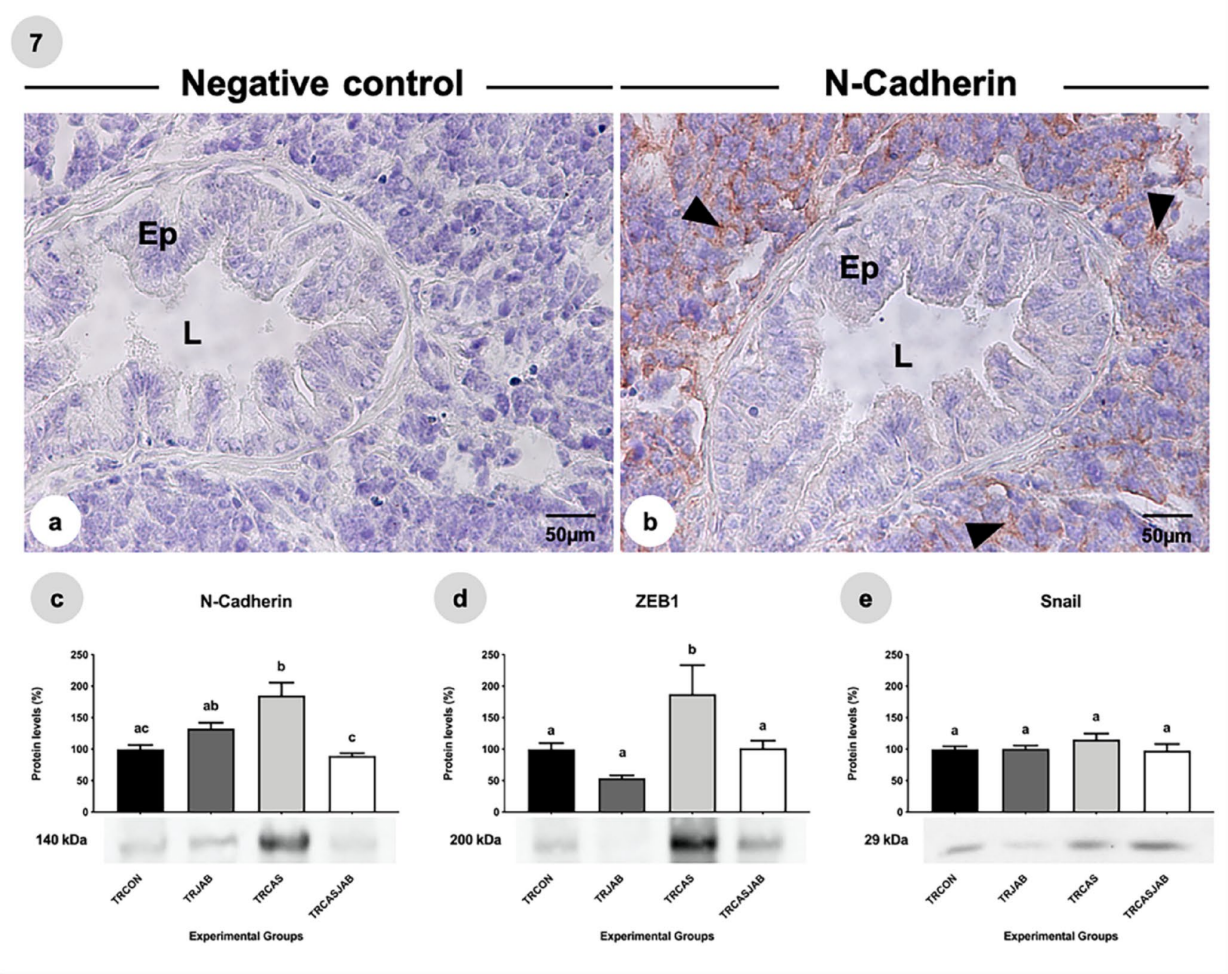
Analysis of the expression of EMT markers/mediators in poorly differentiated tumors from the experimental groups. **A**–**B** Representative photomicrographs of the negative control (**A**), in which the sections were not incubated with the primary antibody, and N-Cadherin immunostaining (**B**). Antigen reactivity was exclusively detected in tumor cells and is indicated by black arrowheads. **C**–**E** Protein levels of N-Cadherin (**C**) and the transcription factors Zinc-finger E-box binding homeobox (ZEB1) (**D**) and Snail (**E)** as determined by Western Blotting. Values are expressed as mean ± standard error of mean in relation to the protein levels of the TRCON group. Different lowercase letters indicate statistically significant differences in the comparison between two experimental groups (P < 0.05). Abbreviations: Ep: epithelium; L: lumen

N-Cadherin immunolabeling in poorly differentiated PCa revealed positive staining of this marker in the sheets of tumor cells, but not in the epithelium of the remaining glands, thus showing that the acquisition of mesenchymal traits is restricted to cells undergoing dedifferentiation and losing the typical glandular architecture (Fig. 7A–B). Furthermore, protein quantification by Western Blotting showed significant higher levels of N-Cadherin in the TRCAS group in relation to the TRCON (185.3% vs 100.0%, respectively; P < 0.001), apart from a clear drop towards control values in the TRCASJAB group (89.55%), thus characterizing a remarkable statistical difference between this group and TRCAS (P < 0.001) (Fig. 7C; Table 3). In agreement with these findings, total protein levels of ZEB1 showed a similar behavior, with enhanced values after castration (187.3% in the TRCAS group vs. 100.0% in the TRCON; P < 0.05) and decreased expression in relation to ADT per se when JPE was associated with this approach (101.5% in the TRCASJAB group; P < 0.05 as compared to TRCAS) (Fig. 7D; Table 3). In spite of these results, no significant differences in Snail expression were observed across the experimental conditions (Fig. 7E; Table 3).

Collectively, these findings indicate that JPE treatment contributed to mitigate some of the harmful changes induced by ADT in the microenvironment of TRAMP mice poorly differentiated tumors. Specifically, administration of the extract was associated with enhanced preservation of typical prostatic glandular architecture, surrounded by a fibromuscular layer primarily composed of smooth muscle cells, as demonstrated by αSMA periacinar staining. This was probably fostered by the remarkable effect of JPE in preventing the acquisition of mesenchymal traits by tumor cells after castration, as shown by the reduced N-Cad and ZEB1 protein levels.

### JPE administration in an androgen-deprived setting enhances TGF-β signaling by modulating the expression and/or activation of ligands and receptors

**Fig. 8:**
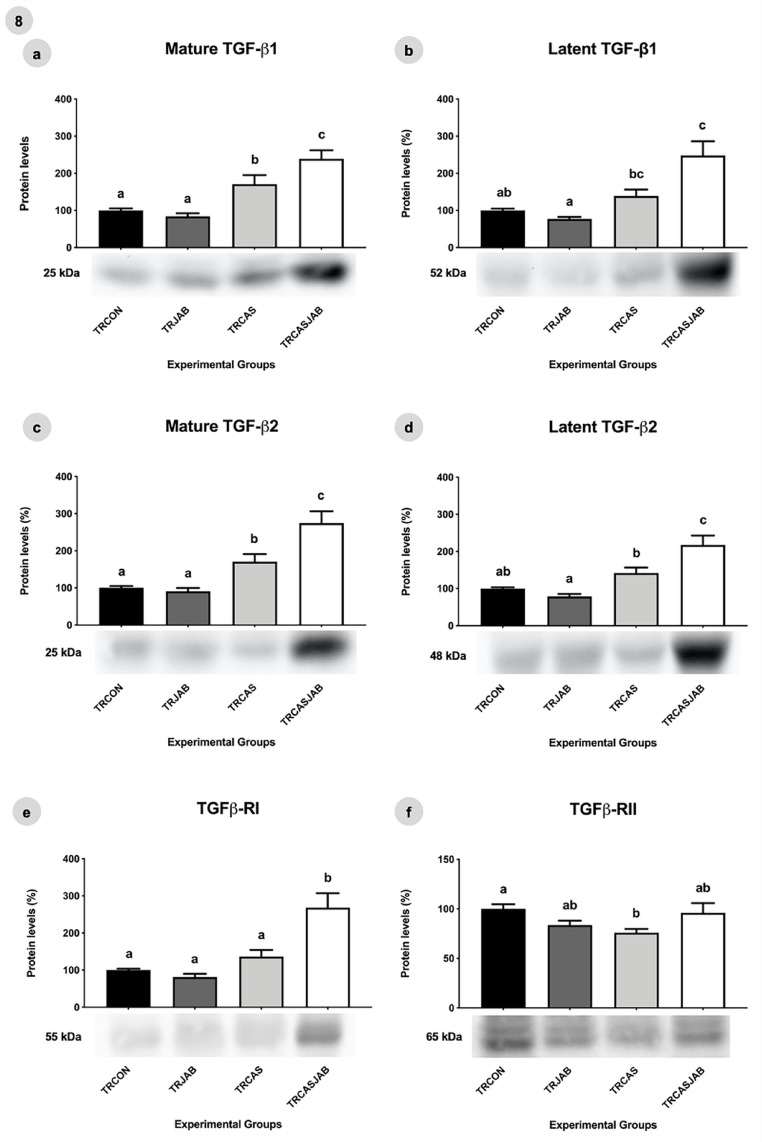
Analysis of the expression of transforming growth factor beta (TGF-β) signaling mediators in poorly differentiated tumors from the experimental groups. **A**–**F** Protein levels of mature (**A**) and latent (**B**) TGF-β1 isoform; mature (**C**) and latent (**D**) TGF-β2 isoform and TGF-β receptors type I (**E**) and II (**F**), as determined by Western Blotting. Values are expressed as mean ± standard error of mean in relation to the protein levels of the TRCON group. Different lowercase letters indicate statistically significant differences in the comparison between two experimental groups (P < 0.05)

TGF-β family includes a series of multifunctional peptides and, in mammalian cells, is composed by the isoforms TGF-β1, TGF-β2 and TGF-β3, which regulate proliferation, apoptosis, differentiation and migration processes. It is widely known that TGF-β remains stored in the ECM in a latent form and is converted to a mature and active state through mechanisms involving both enzymatic and non-enzymatic actions (Tie et al. 2022). TGF-β also mediates EMT by stimulating the acquisition of mesenchymal traits in tumor cells (Yang et al. 2015), prompting us to assess the protein levels of TGF-β1, TGF-β2, and their receptors, TGFβ-RI and TGFβ-RII, in tumors from TRAMP mice across our experimental groups (Fig. 8; Table 3).

Both TGF-β isoforms were detected in the latent and mature stages, showing molecular weights of 52 and 25 kDa for TGF-β1, and 48 and 25 kDa for TGF-β2, respectively. Results demonstrated increased protein levels for both TGF-β1 and TGF-β2 after ADT and, remarkably, upon its association with JPE administration. Specifically, the TRCAS group showed 170.6% of the mature TGF-β1 levels expressed in the TRCON (P < 0.05), whereas the TRCASJAB group demonstrated values of 238.8% (P < 0.0001 and P < 0.05 as compared to TRCON and TRCAS, respectively) (Fig. 8A; Table 3). Considering mature TGF-β2, TRCAS and TRCASJAB groups characterized protein levels of 170.7% and 274.7% (P < 0.05 and P < 0.01 in relation to TRCON and TRCAS, respectively) (Fig. 8C; Table 3). Interestingly, this behavior was less evident for latent TGF-β isoforms, since the only significant difference was registered between latent TGF-β2 expression levels in TRCASJAB (217.7%) and TRCAS groups (142.1%) (P < 0.01) (Fig. 8B and D; Table 3). At the same time, TGF-β receptors showed a dual response upon ADT and its association with JPE treatment. In this sense, while TGFβ-RI showed no changes in its expression in the TRCAS group (136.6% vs. 100.0%; P > 0.05 in relation to TRCON), TGFβ-RII protein levels were significantly decreased after ADT (75.92% vs. 100.0%; P < 0.05 in relation to TRCON) (Fig. 8E and F; Table 3). On the other hand, in the TRCASJAB group, we registered a remarkable raised expression of TGFβ-RI (268.5%; P < 0.0001 as compared to both TRCON and TRCAS groups), with no significant changes in TGFβ-RII protein levels (96.13%; P > 0.05 in relation to TRCON and TRCAS groups) (Fig. 8E and F; Table 3). Overall, these differences yielded a 1.6 fold increase in TGFβ-RI/TGFβ-RII ratio upon JPE association with ADT. Finally, JPE administration in a hormone-naïve scenario did not lead to any significant differences in TGF-β receptors or isoforms expression (Fig. 8A–F; Table 3).

Altogether, these findings pointed out that JPE administration combined with ADT stimulates the expression and activation of TGF-β isoforms. Furthermore, they also suggest an increased activation of this signaling pathway in the TRCASJAB group, considering the enhanced relative expression of TGFβ-RI/TGFβ-RII and the critical role of these receptors in modulating the magnitude and specificity of the signaling cascade (Micalizzi et al. 2010b).

## Discussion

We have recently assessed the effects of JPE in hormone-refractory lesions from TRAMP mice dorsolateral prostate (Rezende et al. 2025). In the study herein, we further investigated the response of poorly differentiated tumors from this transgenic model to the association of JPE and ADT, considering literature reports on their distinct origin compared to in situ well-differentiated adenocarcinomas (Huss et al. 2007; Chiaverotti et al. 2008). Present results revealed that hormonal ablation led to enhanced aggressiveness of poorly differentiated tumors in TRAMP mice. These findings are consistent with previous reports, according to which, in some TRAMP mice subpopulations, castration can synchronize and stimulate the development of these tumors from previously quiescent hormone-refractory lesions (Gingrich et al. 1997; Kaplan-Lefko et al. 2003; Johnson et al. 2005; Tang et al. 2008).

Previous studies investigating the isolated chemopreventive potential of plant-derived compounds on CaP progression in the TRAMP model have reported that the effects on primary and/or metastatic tumor development are more pronounced when the intervention is initiated earlier and maintained for a longer duration (Gupta et al. 2001; Adhami et al. 2009; Shukla et al. 2012, 2014). In this sense, Kim et al. (2014) evaluated the chemopreventive action of a green-tea extract on disease progression in TRAMP mice between 12 and 32 weeks-old and found that all controls presented distant metastasis, including in lymph nodes and lungs, whereas only 13% of treated mice showed this type of lesion. On the other hand, Conlon et al. (2015) verified increased frequency of premalignant lesions in 16 week-old TRAMP mice receiving a 10% tomato powder diet during 8 weeks when compared to controls treated with conventional diet. According to the authors, these data may be either due to the age at which the study was initiated, to the lycopene concentration in the diet or even to the duration of its administration (Conlon et al. 2015). Furthermore, Rowles et al. (2020) also investigated the effects of 10% tomato powder or lycopene diets in the TRAMP model, but in androgen-deprived hosts. By offering the diet to the experimental mice from the onset of castration (12–13 weeks) until 30 weeks-old, the authors observed no differences in moderate disease and histologically confirmed malignancy, in spite of former registers pointing to the ability of similar diets in delaying the development of hormone-responsive PCa in this model (Konijeti et al. 2010; Pannellini et al. 2010; Tan et al. 2017; Rowles et al. 2020). Altogether, these findings suggest that the attenuation of natural compounds chemopreventive effects in late interventions in TRAMP mice may be related with the hormone-refractory phenotype intrinsic to the model with advancing age.

Indeed, Kaplan-Lefko et al. (2003) mapped the development of lesions in the TRAMP model under different androgen reliance settings and found that castration at 12 weeks-old had a deleterious impact in the course of the disease. In this sense, the authors reported that around 60% of castrated mice developed poorly differentiated macroscopic tumors, apart from showing higher incidence of lymphatic and pulmonary metastasis in relation to controls, thus reinforcing previous registers of the literature (Gingrich et al. 1997; Kaplan-Lefko et al. 2003). Similarly, Johnson et al. (2005) verified that delaying the castration in TRAMP model led to the development of less aggressive tumors, enhancing mice lifespan. In agreement with these data, we have previously demonstrated that early and long-term ADT in TRAMP mice, which was achieved through orchiectomy and flutamide administration from 12 to 22 weeks-old, had deleterious effects on PCa progression, particularly when it comes to angiogenesis regulation, thereby impairing the otherwise beneficial actions of JPE. In this setting, the combination of the extract with ADT led to poor disease outcomes, such as decreased survival, lower proportion of tumor-free mice and enhancement of proliferative lesions (Rezende et al. 2025), suggesting potentially harmful effects of extending this combined approach. Based on these findings, in the study herein, we chose to limit the duration of JPE association with ADT to 6 weeks (from 16 to 22-weeks-old). Consistent with the abovementioned data, we found that following ADT, achieved through castration and enzalutamide treatment, poorly differentiated macroscopic tumors, previously prevalent in the ventral prostate, spread to other prostatic lobes, occupying them in statistically similar proportions. On the other hand, results herein are unique since they demonstrate that JPE polyphenols had chemopreventive potential even in advanced and poorly differentiated tumors with CRPC traits. This was evidenced by the increased proportion of tumor-free mice in the TRCASJAB group, which overcame the rate of tumors comprising two or all the prostatic lobes. This finding may be linked to prior evidence reporting that anthocyanins suppress cancer cell proliferation, promote cell cycle arrest and trigger apoptosis in PCa cells in vitro, effects that may be partially attributable to their pro-oxidant activity in tumor cells, which is mediated by the generation of reactive oxygen species (ROS) (Reddivari et al. 2007; Bin Hafeez et al. 2008; Long et al. 2013; León-González et al. 2015; Ha et al. 2015; Sorrenti et al. 2015). Finally, our results also showed that metastasis development did not parallel these effects, considering there were no changes in the incidence of these lesions following androgen ablation per se or associated with JPE. This result is in agreement with registers from Kaplan-Lefko et al. (2003), who reported metastasis occurrence even in castrated TRAMP mice displaying prostatic regression and no detectable primary tumors, thereby showing that metastasis development in this model is uncoupled from the emergence of poorly differentiated prostatic primary lesions.

Our results also revealed, for the first time to our knowledge, that JPE is capable of reducing AR expression in poorly differentiated tumors from castrated TRAMP mice. This finding is in line with previous registers by our group, which reported lower AR protein levels in ventral prostate microenvironment of elderly non-transgenic mice following JPE administration (Lamas et al. 2020). However, we did not observe such a decrease in AR levels when studying the effects of the same dose of JPE in TRAMP mice dorsolateral prostate under hormone-naïve and ADT settings (Rezende et al. 2025). Taken together, these data suggest that, besides the dose applied, several other factors, such as experimental model, prostatic lobe, hormonal *status* or disease stage (in situ lesions vs. poorly differentiated tumors) may interfere on prostatic response to JPE treatment. Regardless of this fact, it is well known that AR displays an essential role in PCa development and progression from hormone-dependent initial lesions towards CRPC, which is a stage characterized by overexpression and inappropriate activation of signaling pathways mediated by this receptor, even in a milieu with extremely low levels of androgens (Balk and Knudsen 2008; Pu et al. 2017; Li et al. 2025). Therefore, in the study herein, the raised expression of AR following orchiectomy and chemical castration with enzalutamide points to the effective development of tumors with phenotypical CRPC traits in the TRCAS group, a result also supported by the similar rate of cell proliferation in relation to the TRCON group, as demonstrated by the statistically equal protein levels of PCNA. Altogether, these data reinforce that the chemopreventive effects of JPE on macroscopic tumor incidence and AR expression observed in the TRCASJAB group indeed occurred within a CRPC microenvironment, thereby contrasting with earlier reports suggesting that JPE’s beneficial actions depend on androgen signaling (Kido et al. 2022; Rezende et al. 2025). Remarkably, in our previous study conducted by Rezende et al. (2025), we registered that JPE had a protective effect over the development of poorly differentiated tumors only in a hormone-naïve setting and after long-term treatment. Based on this, it is worth keeping in mind that we previously used flutamide to obtain androgen deprivation, a drug which has been proved to be less efficient in comparison to enzalutamide in both preclinical and clinical settings, considering its lower affinity and limited ability to prevent AR nuclear translocation as well as the capacity of acting as an agonist of the receptor under certain circumstances, such as under AR overexpression and mutations (Culig 2017; Kawahara et al. 2017; Rice et al. 2019; Iguchi et al. 2020; Uemura et al. 2022).

At the same time, there is a growing body of evidence reporting the role of estrogens in PCa development and progression, including their contribution to EMT occurrence in tumor cells in vitro (Bonkhoff 2018; Gadkar et al. 2022). In this sense, it is known that the natural course of PCa development during aging is associated with a shift in the expression ratio between ERβ and ERα towards the latter, thereby fostering its oncogenic actions, which include induction of cell cycle progression, cell proliferation and survival, as opposed to ERβ antiproliferative and pro-apoptotic effects (Omoto and Iwase 2015; Belluti et al. 2023; Wu et al. 2025). In agreement, it has also been reported that while ERβ is partially lost, estrogenic signaling mediated by both ERα and orphan receptors progressively emerge during transition to CPRC, aiding tumor cells to bypass the AR requirement for their growth (Bonkhoff 2018). These data prompted us to investigate the protein expression of both estrogen receptors in the poorly differentiated tumors of the experimental groups.

As previously reported by others, results showed a remarkable raise in estrogenic signaling following ADT with an AR antagonist, whereas the association of JPE treatment with androgen depletion drove this signaling towards ERβ actions, which are widely known by their primary protective role against PCa progression (Ślusarz et al. 2012; Wu et al. 2017; Bonkhoff 2018; Qu et al. 2020). Interestingly, these tumor-suppressor roles have been attributed to ERβ1 (Ma et al. 2026), which is the specific splice variant detected by the antibody used in this study. In this sense, Bektic et al. (2004) registered that genistein, a polyphenol largely found in soy byproducts, promoted ERβ signaling in LNCaP cells, thereby inducing decreased AR protein concentration and PSA expression in this androgen-dependent PCa cell line. Consistently, other literature studies have reported the antagonism between ERβ and AR signaling also in the ventral prostate of mice. In this scenario, treatment with a ERβ-selective agonist increased the expression of this receptor while suppressing AR functions through repression of its coactivators, regardless of the androgen reliance of the disease (Wu et al. 2017, 2025). In agreement, in the TRAMP model, ERβ knockout resulted in a twofold increase in the incidence of poorly differentiated tumors in relation to wild-type mice, whereas ERα knockout had almost no incidence of such lesions (Ślusarz et al. 2012).

In line with the findings herein, epidemiological studies with patients fed with dietary phytoestrogens, especially soy-derived ones, reported significant association with reduced risk for CaP development (He et al. 2015; Zhang et al. 2016). Indeed, it is known that the phenolic ring structure which characterizes polyphenols enables their interaction with both ERα and ERβ, blocking or altering the effects of 17β-estradiol and thus supporting their classification as selective ER modulators (SERMs) (Cipolletti et al. 2018). According to Thelen et al. (2014), such effects may be associated with the specific interactions of these bioactive compounds with ERβ, promoting upregulation of this receptor and counteracting the expression of AR and its coactivators, thus leading to PSA decline in the CRPC setting. Indeed, Mentor-Marcel et al. (2001) reported a significant reduction in poorly differentiated tumor incidence in TRAMP mice fed with a genistein-enriched diet, whereas no effects were observed at lower grade disease stages. However, Ślusarz et al. (2012) later found a significant decrease in the incidence of well- but not poorly differentiated carcinoma, a difference that the authors partially attributed to the earlier completion of genistein administration, thus suggesting that the protective role of this phytoestrogen against prostatic tumorigenesis is greater in the early stages of the disease. Surprisingly, the authors also reported that the functional integrity of both ERα and ERβ was required for the observed preventive effects of genistein (Ślusarz et al. 2012). Collectively, these results pointed to differential effects of phytoestrogens in TRAMP mice depending on disease stage and suggested that these differences may rely on the balance between the expression and subcellular localization of ER subtypes in each phase, with nuclear ERβ prevailing in PIN and well-differentiated PCa and ERα being mostly expressed in the nucleus of poorly differentiated tumor cells (Ślusarz et al. 2012). Taking this into account, our results showing a shift on the balance between ERα and ERβ expression following JPE and ADT association led us to conclude that the extract components favored estrogenic signaling mediated by ERβ, thereby opposing AR action towards CRPC progression and attenuating the aggressiveness of poorly differentiated tumors in TRAMP mice.

According to Kaplan-Lefko et al. (2003), EMT consists in a mechanism by which carcinomas tend to lose their epithelial traits throughout their development to acquire a mesenchymal phenotype with higher metastatic potential. In this sense, when studying tumor progression in the TRAMP model, these authors registered that PIN and well-differentiated adenocarcinoma lesions maintained the expression of E-Cadherin, whereas more advanced stages, such as poorly differentiated tumors, showed reduction or even absence of this adhesion molecule (Kaplan-Lefko et al. 2003). In the latter lesions, E-Cadherin expression was reported to be restricted to the persistent glands scattered between tumor cells (Kaplan-Lefko et al. 2003), thus resembling our observations herein. At the same time, the authors mentioned that the acquisition of the neuroendocrine traits found in these tumors is an intrinsic and late event during CaP progression in the TRAMP model, which occurs by a mechanism of phenotypic plasticity referred to as epithelial-to-neuroendocrine transition (ENT) (Kaplan-Lefko et al. 2003). In parallel with these observations, Wang et al. (2024) reported that the process of epithelial to mesenchymal plasticity (EMP) comprises the mechanisms of EMT and mesenchymal-to-epithelial transition (MET) and can not be considered as binary. In this sense, it was proposed that cancer cells transit between epithelial and mesenchymal phenotypes to adapt themselves to the new conditions during tumor progression, being able to assume even an intermediate phenotype between the two extremes, which could render them increased invasive and metastatic potential (Wang et al. 2024). Based on these observations, we can hypothesize that, in the poorly differentiated tumors of our experimental groups, the decrease of E-Cadherin expression perhaps is not an essential event for CaP progression, since at this stage tumor cells might be in an intermediate neuroendocrine differentiation state in which the expression of this adhesion molecule have already diminished. Apart from that, the occurrence of similar protein levels for E-Cadherin across all experimental conditions may also be related with the absence of significant differences in metastases incidence, considering that this parameter may indicate similar degrees of adhesion between tumor cells and the surrounding extracellular matrix (ECM) in the different groups.

Regarding αSMA reactivity in the poorly differentiated tumors, we found interesting results, especially when it comes to its localization in the periacinar layer. In this sense, while TRCAS tumors were characterized by remarkable loss of the smooth muscle layer, thereby indicating drastic changes in glandular architecture following ADT, the association of this approach with JPE in the TRCASJAB group led to a significant increase in periacinar αSMA positivity. These data suggest that, although not being able to prevent or delay PCa progression towards a poorly differentiated phenotype, JPE did contribute for prostatic morphology maintenance, leading to enhanced preservation of the acinar structure, a result that is probably associated with the lower aggressiveness of tumors in the TRCASJAB group. At the same time, data from vimentin expression analysis showed no changes on its immunolabeling frequency or protein levels in the tumors from the different experimental groups. Altogether, these findings are relevant, since they indicate that changes in tumor milieu upon JPE and ADT combination were not associated with vimentin overexpression, an event which could indicate the occurrence of stromal cells transdifferentiation into reactive cell types, such as myofibroblasts and cancer associated fibroblasts (CAFs), which are characterized by the coexpression of these markers (Tuxhorn et al. 2002; Ting et al. 2015).

Indeed, several literature reports pointed out the beneficial effects of flavonoids in PCa-associated tumor microenvironment, counteracting the induction of reactive stromal phenotypes able to secrete growth factors and pro-tumor cytokines (Gray et al. 2014; Ting et al. 2015, 2016). According to Ting et al. (2015), who performed in vitro experiments with the flavonoid silibinin, such an antagonism would include both direct effects on stromal cells as well as indirect actions on the secretory activity of tumor cells, thus interfering with the synthesis of molecular mediators of CAF recruitment, such as TGF-β2. The same authors later found that diet supplementation with this flavonoid led to decreased expression of CAF biomarkers, such as vimentin and αSMA, in mice bearing TRAMP-derived allografts (Ting et al. 2016). Finally, it was also registered that silibinin inhibited the capacity of these reactive cells to induce EMT in CaP cell cultures, as demonstrated by restoration of E-Cadherin levels in tumor cells (Ting et al. 2016).

Similarly, the results herein demonstrated the ability of JPE polyphenols in counteracting the acquisition of mesenchymal markers, such as N-Cadherin, by tumor cells in TRAMP mice undergoing ADT. In agreement with our findings, Pu et al. (2017) registered that castration led to raised protein expression of this molecule in prostatic tumors in the TRAMP model, even though such authors have also registered simultaneous decrease in E-Cadherin levels, as opposed to our results. N-Cadherin is well known for being aberrantly expressed in several malignant diseases, including PCa, and for its association with tumorigenesis, metastases and poor outcomes, in such a manner that its silencing has therapeutic potential to suppress primary or metastatic tumor growth (Nakazawa and Kyprianou 2017; Cao et al. 2019; Jolly et al. 2021). Interestingly, N-Cadherin has been shown to promote invasion of breast cancer cells even under sustained E-Cadherin expression, thereby overcoming the adhesive properties of this molecule (Nieman et al. 1999). Furthermore, Wang et al. (2024) reported that raised ZEB1 expression is involved in the positive regulation of N-Cadherin, whereas Sun et al. (2012) registered that ADT induces EMT in both normal and neoplastic prostate tissues through mechanisms involving ZEB1 overexpression. Indeed, our results showed that the lower levels of N-Cadherin in the TRCASJAB group were paralleled by decreased protein expression of ZEB1 following JPE treatment associated with ADT, thus suggesting a possible regulatory effect.

Several studies have also highlighted the protective role of ERβ in maintaining the epithelial phenotype in the prostate, as it promotes E-Cadherin expression by acting on its gene promoter, thereby attenuating the aggressive behavior associated with EMT (Mak et al. 2010; Christoforou et al. 2014). Accordingly, treatment of PCa cells with TGF-β or their exposure to hypoxic conditions, which are well-known EMT inducers, resulted in the acquisition of a spindle-like morphology, loss of E-Cadherin, and increased expression of vimentin and N-Cadherin, phenotypic changes associated with decreased ERβ expression, but not ERα (Mak et al. 2010; Christoforou et al. 2014). Thus, based on the results regarding the above markers, we hypothesize that, in an androgen-deprivation setting, JPE bioactive compounds exerted positive effects on tumor milieu, favoring the maintenance of stromal smooth muscle cells in a quiescent, contractile phenotype rather than a secretory and reactive one. Furthermore, such phenotypic modulation in stromal cells may have negatively influenced EMT occurrence in tumors, thereby suggesting that inhibition of this process represents one of the mechanisms contributing to the beneficial effects of JPE. Still, we raised the possibility of ERβ involvement on this mechanism, considering its antagonistic role on EMT modulation by means of the crosstalk with TGF-β signaling. This scenario led us to investigate the TGF-β pathway in our experimental groups.

TGF-β stands out as the most well-known inducer of EMT due to its ability to suppress E-Cadherin expression by upregulating its repressors ZEB1, Snail and Twist1 (Derynck et al. 2001; Cao and Kyprianou 2015; Lin and Wu 2020; Lee and Massagué 2022). Additionally, a remarkable paradox regarding this signaling pathway is TGF-β dual role during CaP development, whereby it counteracts cell proliferation and induces apoptosis in the early disease stages and promotes metastasis in later steps (Cao and Kyprianou 2015; Adekoya and Richardson 2020; Baba et al. 2022; Lee and Massagué 2022). In spite of this consensus, several authors reported that the final biological effects of TGF-β pathway are not absolute and largely depend on the cell context (Pu et al. 2009, 2017; Micalizzi et al. 2010b). Among the proposed mechanisms for the loss of TGF-β growth-suppressor actions, changes in expression and functional integrity of its receptors are highlighted, especially when it comes to TGFβ-RII, which initiates the signaling cascade (Williams et al. 1996; Zhang et al. 2005; Mishra et al. 2014; Zhou et al. 2018). This includes Smad family members in the canonical pathway, and molecules involved in non-canonical signaling cascades, such as phosphoinositide 3-kinase(PI3K)/AKT and p38 mitogen-activated protein kinase (MAPK) (Kretzschmar 2000; Derynck et al. 2001; Shi et al. 2022). In this sense, loss of TGFβ-RII expression through various mechanisms has been reported to be associated with the development of several cancer types, as well as with increased PCa aggressiveness (Williams et al. 1996; Kretzschmar 2000; Zhang et al. 2005). In agreement, studies carried out with genetically modified TRAMP mice expressing a dominant-negative TGFβ-RII (DNTGFβ-RII) in the prostatic epithelium, thereby abolishing cell response to the growth factor, demonstrated that loss of TGF-β signaling may also induce EMT (Pu et al. 2009, 2017). At the same time, Nakazawa and Kyprianou (2017) reported that only the non-canonical TGF-β pathway is able to induce EMT, whereas the growth-inhibitory actions of this molecule are mediated by the Smad-dependent canonical signaling.

Results herein demonstrated that ADT led to reduced levels of TGFβ-RII, which may have accounted for the raised expression of mature TGF-β isoforms observed in the TRCAS group. However, JPE administration following ADT not only promoted TGF-β accumulation but also increased the TGFβ-RI/TGFβ-RII ratio, suggesting that this signaling pathway remained functionally intact, probably through the canonical pathway, as evidenced by the its tumor-suppressive effects on PCa aggressiveness. Finally, according to Micalizzi et al. (2010a), only EMT occurrence and enhanced TGF-β signaling are not sufficient for metastasis development, indicating the involvement of other molecular mediators in this process. Altogether, these findings may help to explain our observations of EMT occurrence and enhanced TGF-β signaling as decoupled events in the TRCAS and TRCASJAB groups, respectively, as well as the similar incidence of metastases observed in these experimental groups.

In agreement with our results, Suenaga et al. (2008) registered that resveratrol, a stilbene polyphenol, increased TGF-β2 expression both at gene and protein levels in lung cancer cell lines, thus leading to autocrine activation of Smad signaling and beneficial effects on tumor cells. Furthermore, the authors reported that the influence of resveratrol on TGF-β signaling may have involved its action as a phytoestrogen, that is, a molecule structurally similar to 17β-estradiol with ability to act as an agonist of estrogen receptors (Suenaga et al. 2008; Nanashima et al. 2018; Sohel et al. 2022). Specifically, antitumor effects of phytoestrogens have been preferentially attributed to their interactions with ERβ, which is overexpressed in various PCa stages in the TRAMP model, including poorly differentiated tumors as those analyzed herein (Ślusarz et al. 2012; Bonkhoff 2018; Qu et al. 2020). In particular, Nanashima et al. (2018) demonstrated in vitro the ERβ-mediated phytoestrogenic activity of blackcurrant anthocyanins, either isolated or as part of a whole extract, by means of their higher binding affinity to this receptor in relation to ERα and ability to induce its transcriptional activity. Importantly, these anthocyanins included cyanidin-3-glucoside (C3G), cyanidin-3-rutinoside (C3R), delphinidin-3-glucoside (D3G) and delphinidin-3-rutinoside (D3R) (Nanashima et al. 2018), some of which are also present in the JPE. Furthermore, Liu et al. (2019) reported that cyanidin-3-glucoside also enhanced ERβ signaling in vivo, thereby leading to cell cycle arrest and apoptosis induction, which colectively resulted in decreased tumor growth in a murine model of melanoma.

Consistent with the aforementioned findings, our results showed enhanced TGF-β signaling in TRAMP tumors following treatment with anthocyanin-rich JPE under androgen-deprived conditions, an effect which was paralleled by an increased ERβ/ERα ratio, suggesting prevalence of ERβ-mediated estrogenic signaling. Moreover, based on the previously reported negative regulation of AR expression by ERβ following phytoestrogen administration (Thelen et al. 2014), and considering the well-documented antagonism between androgens and TGF-β (Lucia et al. 1998; Dallas et al. 2005; Danielpour 2005), it can be speculated that TGF-β stimulation observed in the TRCASJAB group may have resulted from the reduced levels of AR orchestrated by this estrogen receptor. At the same time, Silva et al. (2018) reported the unique role of ERβ, but not ERα, in the modulation of phenotypic plasticity in CRPC cell lines toward an epithelial state by inhibiting N-Cadherin and promoting E-Cadherin expression. In line with this, ERβ has been shown to inhibit metastatic potential of triple-negative breast cancer cells through its suppressive effects on ZEB1 and N-Cadherin, along with upregulation of E-Cadherin (Song et al. 2017). Taken together, these findings led us to hypothesize that, in the TRCASJAB group, a similar effect of JPE-induced ERβ signaling may have suppressed ZEB1 expression and, consequently, N-Cadherin levels in poorly differentiated tumors, thereby counteracting EMT and reducing PCa aggressiveness.

Collectively, the abovementioned information led us to assume that the observed effects of JPE administration on CRPC tumor cells from the TRCASJAB group may have branched from at least two action trends of its polyphenolic compounds, especially anthocyanins. Firstly, in line with previous registers, these molecules may have directly interfered with essential cellular mechanisms involved in tumor progression, thereby inhibiting cell proliferation, inducing DNA damage and apoptosis or impairing migration and invasion, either through direct actions in the expression of EMT markers or by regulating the activity of transcription factors, such as ZEB1 and Snail (León-González et al. 2015; Burton et al. 2015; Amawi et al. 2017; Lin et al. 2017; Cipolletti et al. 2018; Liu et al. 2019; de Arruda Nascimento et al. 2022). This would possibily occur through ROS generation and induction of pro-oxidant damage, particularly in the presence of transition metal ions such as copper, which are found at high concentrations in cancer microenvironment, thus helping to explain the selective cytotoxicity of polyphenols toward tumor cells when compared with normal cells, in which they exert antioxidant effects (Hadi et al. 2000; León-González et al. 2015; Amawi et al. 2017). Alternatively, taking into consideration the documented role of cyanidin-3-glucoside, delphinidin-3-glucoside and other anthocyanins as phytoestrogens as well as their high affinity to bind ERβ (Nanashima et al. 2018), we can also suggest that the beneficial effects observed upon JPE administration may have been primarily mediated by the well-known tumor-suppressive actions of this receptor in the CRPC microenvironment. These may include inhibition of survival and proliferation pathways, as well as prevention of migration, invasion and EMT, possibly through fine-tuning TGF-β signaling towards its growth-suppressive roles (Piccolella et al. 2014; Silva et al. 2018; Ma et al. 2026). At the same time, it has been reported that the ultimate effects of polyphenol exposure mainly depend on the ERα/ERβ expression pattern in the target tissue as well as on their relative affinity for each estrogen receptor subtype, which is, in the case of anthocyanins, greater for ERβ (Cipolletti et al. 2018; Nanashima et al. 2018). Thus, based on these data, and considering the marked shift in the expression ratio toward this receptor observed in our study following JPE association with ADT, we postulate that ERβ-mediated signaling represents the most plausible mechanism underlying the observed effects. Furthermore, in line with previous reports (Gehrig et al. 2017), we propose that the bioactive compounds in JPE are acting as subtype-specific SERMs, thus modulating estrogen pathways towards the anticancer actions of ERβ and providing a potential adjuvant therapy to circumvent the development of resistance to enzalutamide through EMT and other mechanisms. Nevertheless, the confirmation of ERβ central role in mediating JPE-associated antitumor effects in this setting requires further studies involving coadministration of the extract with an ERβ antagonist.

## Conclusion

In conclusion, our findings demonstrate, to the best of our knowledge, the first evidence that JPE attenuates the aggressiveness of poorly differentiated CRPC in the TRAMP model by modulating steroid hormone receptors, particularly ERβ, within the tumor microenvironment. In this sense, the extract promoted a shift on estrogenic signaling towards ERβ-mediated pathways, thereby counteracting the harmful effects of ERα and AR that contribute to CRPC progression. Specifically, we postulate that these JPE-induced events under ADT conditions may involve the conversion of TGF-β signaling from a growth-promoting to a tumor-suppressive agent, adding complexity to the actions of this molecule and pointing its role as a potential mediator of the beneficial effects of ERβ. These may include limiting the acquisition of mesenchymal and/or reactive traits by stromal cells, effects likely orchestrated by canonical TGF-β signaling. Collectively, our results highlight the potential of JPE as an adjuvant therapy to ADT and open new avenues for future investigations into the precise molecular mechanisms underlying ERβ and TGF-β actions in this scenario.

## Supplementary Information

Below is the link to the electronic supplementary material.

Supplementary Material 1

## Data Availability

The data that support the findings of this study are not openly available due to reasons of sensitivity and are available from the corresponding author upon reasonable request.
