# Supplementary material for: Jaboticaba peel extract limits castration-resistant prostate cancer aggressiveness by counteracting EMT through steroid hormone and TGF-β signaling modulation
Source: J Mol Histol. 2026 Jun 22;57(4):200. doi: 10.1007/s10735-026-10851-x (PMC13287156; doi:10.1007/s10735-026-10851-x)

**Table S1.** Antigens detected by immunohistochemistry and/or Western Blotting and their respective primary antibodies.

| **Antigens** | **Primary antibodies** | |
| --- | --- | --- |
| **AR** | | Rabbit polyclonal ab74272 (*Abcam*) |
| **E-Cadherin** | | Rabbit monoclonal #3195 (*Cell Signaling Technology*) |
| **ERα** | | Rabbit polyclonal sc-542 (*Santa Cruz Biotechnology*) |
| **ERβ** | | Mouse monoclonal ab187291 (*Abcam*) |
| **N-Cadherin** | | Rabbit monoclonal #13116 (*Cell Signaling Technology*) |
| **PCNA** | | Mouse monoclonal ab29 (*Abcam*) |
| **Snail** | | Rabbit monoclonal #3879 (*Cell Signaling Technology*) |
| **SV40 Large T** | | Mouse monoclonal sc-147 (*Santa Cruz Biotechnology*) |
| **TGF-β1** | | Mouse monoclonal ab64715 (*Abcam*) |
| **TGF-β2** | | Mouse monoclonal ab36495 (*Abcam*) |
| **TGFβ-RI** | | Rabbit monoclonal ab235578 (*Abcam*) |
| **TGFβ-RII** | | Rabbit monoclonal ab259360 (*Abcam*) |
| **Vimentin** | | Rabbit monoclonal ab92547 (*Abcam*) |
| **ZEB1** | | Rabbit monoclonal #70512 (*Cell Signaling Technology*) |

**Table S2.** Comparative analysis of poorly differentiated tumor weight, final body weight and their ratio among experimental groups.

| **Parameter** | **Experimental groups** | | | | | | | |  |
| --- | --- | --- | --- | --- | --- | --- | --- | --- | --- |
|  | **TRCON** | | **TRJAB** | | **TRCAS** | | **TRCASJAB** | |  |
| **Tumor weight (g)*** | | 4.813 ± 1.129^a^ | | 5.113 ± 1.183^a^ | | 4.111 ± 1.079^a^ | | 3.236 ± 1.076^a^ | |
| **Final body weight (g)*** | | 34.29 ± 0.7962^a^ | | 32.86 ± 0.7709^ab^ | | 31.39 ± 0.7216^bc^ | | 29.05 ± 0.6496^c^ | |
| **Tumor / final body weight (%)*** | | 13.44 ± 2.888^a^ | | 14.01 ± 2.909^a^ | | 11.93 ± 3.033^a^ | | 10.14 ± 3.315^a^ | |

Different lowercase letters indicate statistically significant differences among the groups (P<0.05).

Statistical test: * One-way ANOVA (Tukey`s post hoc test).

**
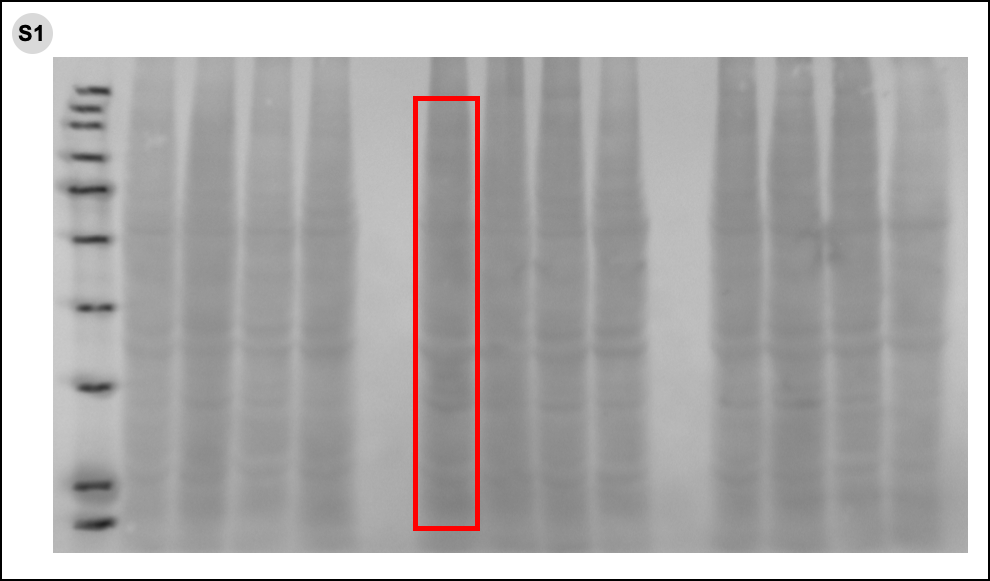
**

**Figure S1.** Representative image of a Western Blotting membrane after Ponceau S staining. Protein loading control was assessed by measuring the optical density of an area similar to the indicated inside the red rectangle, thereby reflecting the total protein content in each sample.

**
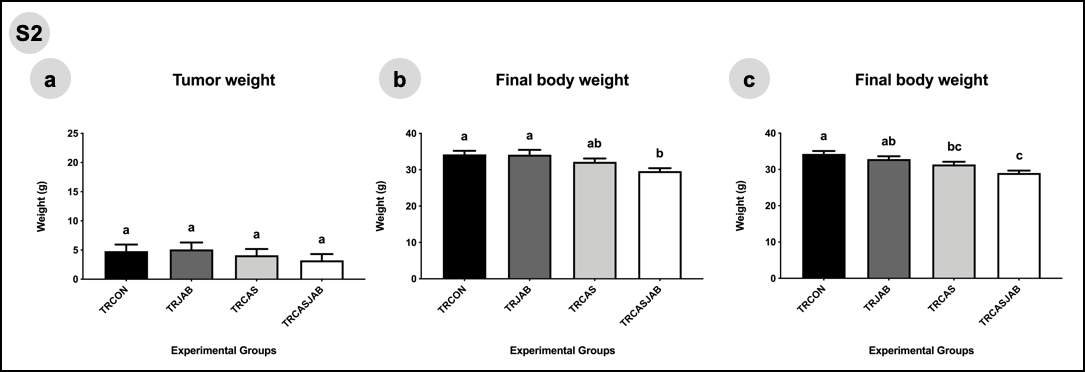
**

**Figure S2.** Analyses of weight-related parameters in TRAMP mice of the experimental groups. Panels indicate poorly differentiated tumor weight (A), final body weight (B) and tumor to final body weight ratio (C). Different lowercase letters indicate statistically significant differences among the groups.

**Western Blotting raw data:**

Each figure depicts representative Western Blotting membranes used to analyze the protein expression of the investigated molecules indicated at the top of the image. The membranes represent two independent experiments including different animals (biological replicates) from the TRCON, TRJAB, TRCAS and TRCASJAB groups and are presented exactly as captured by the image acquisition software. Total protein loading for each sample was normalized using Ponceau S staining, as described in the Methods section. It should be noted that Ponceau S staining may have been performed on a different date from antibody incubation and that some membranes include fewer than three animals per group. Nevertheless, at least four animals per group were included in the analysis of each antigen. For each membrane, the mean densitometric value of the TRCON group was calculated and used as a reference standard (100.0%) to determine relative protein expression levels in the other groups. This approach enabled the integration of raw data obtained from different membranes for the analysis of protein expression of a given antigen.


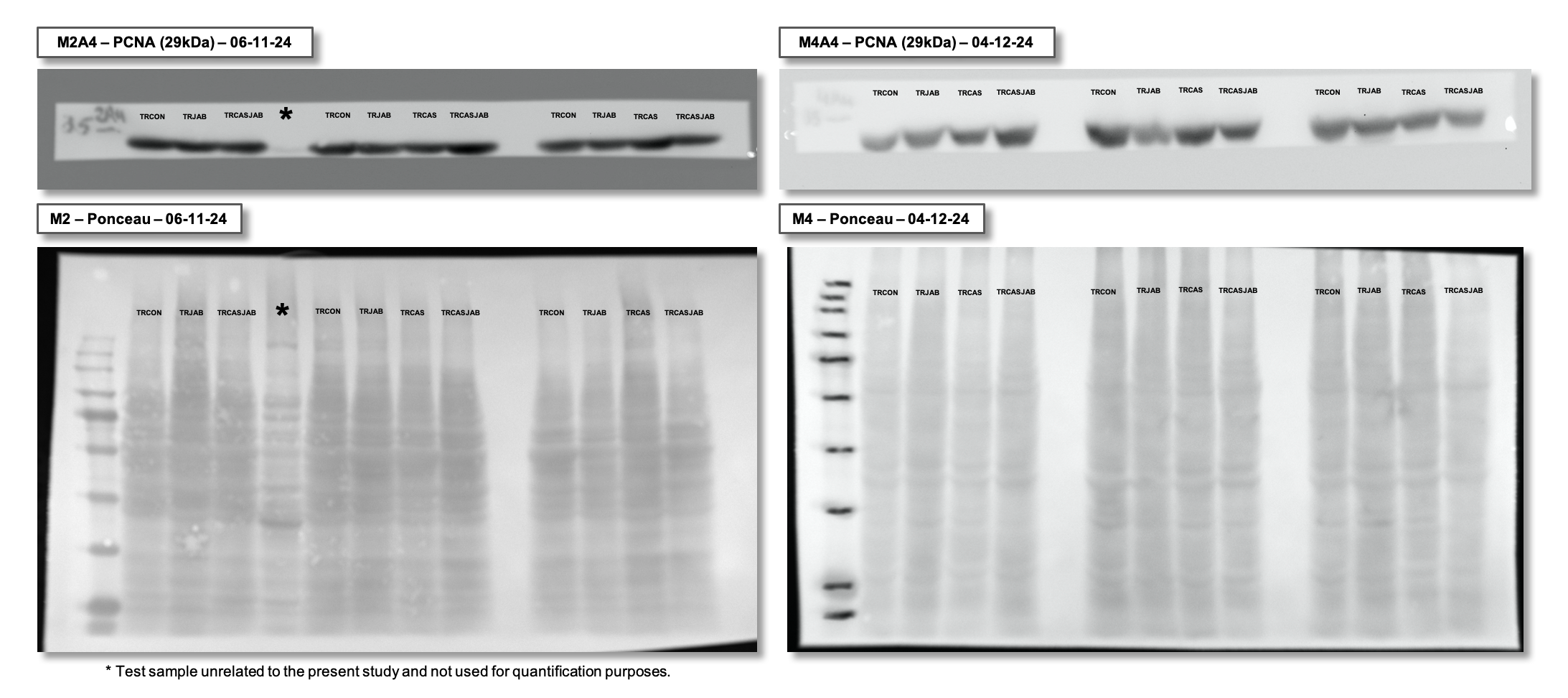

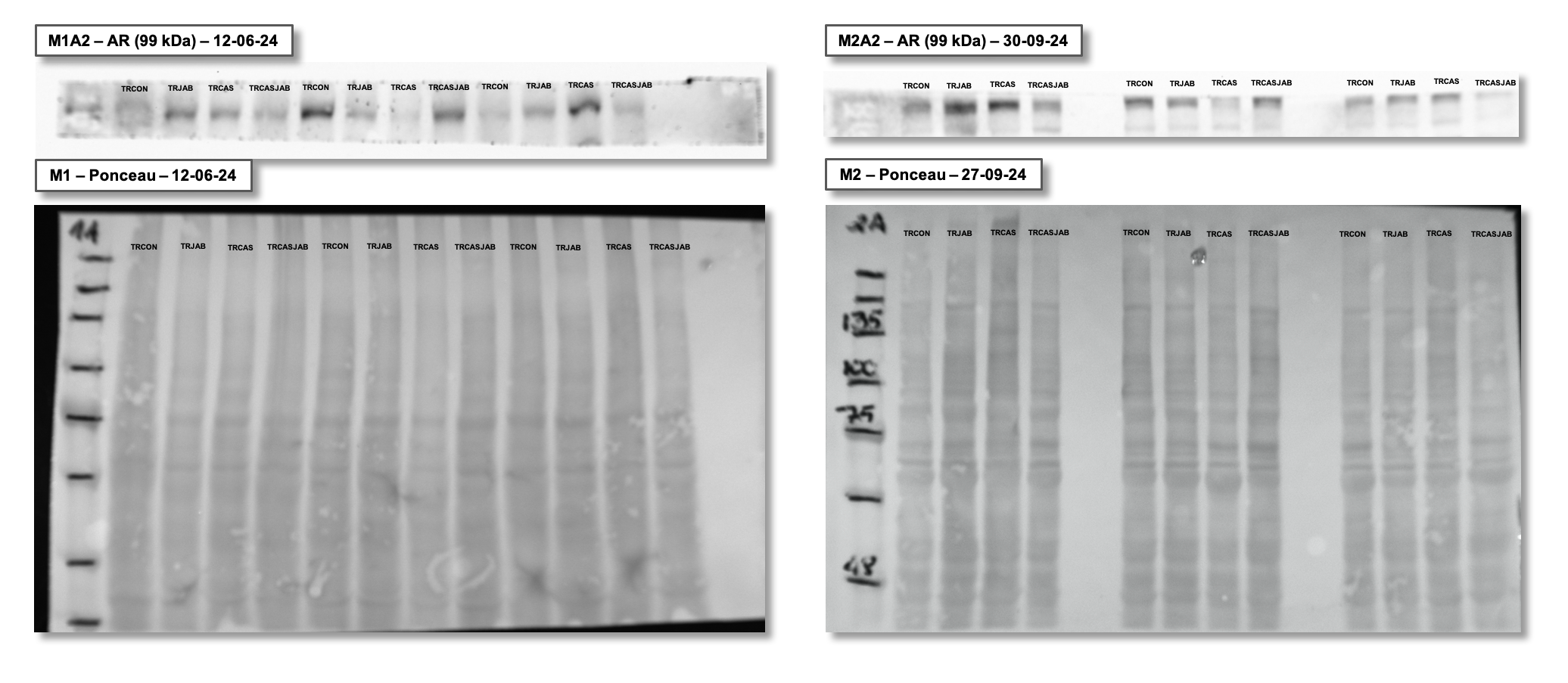


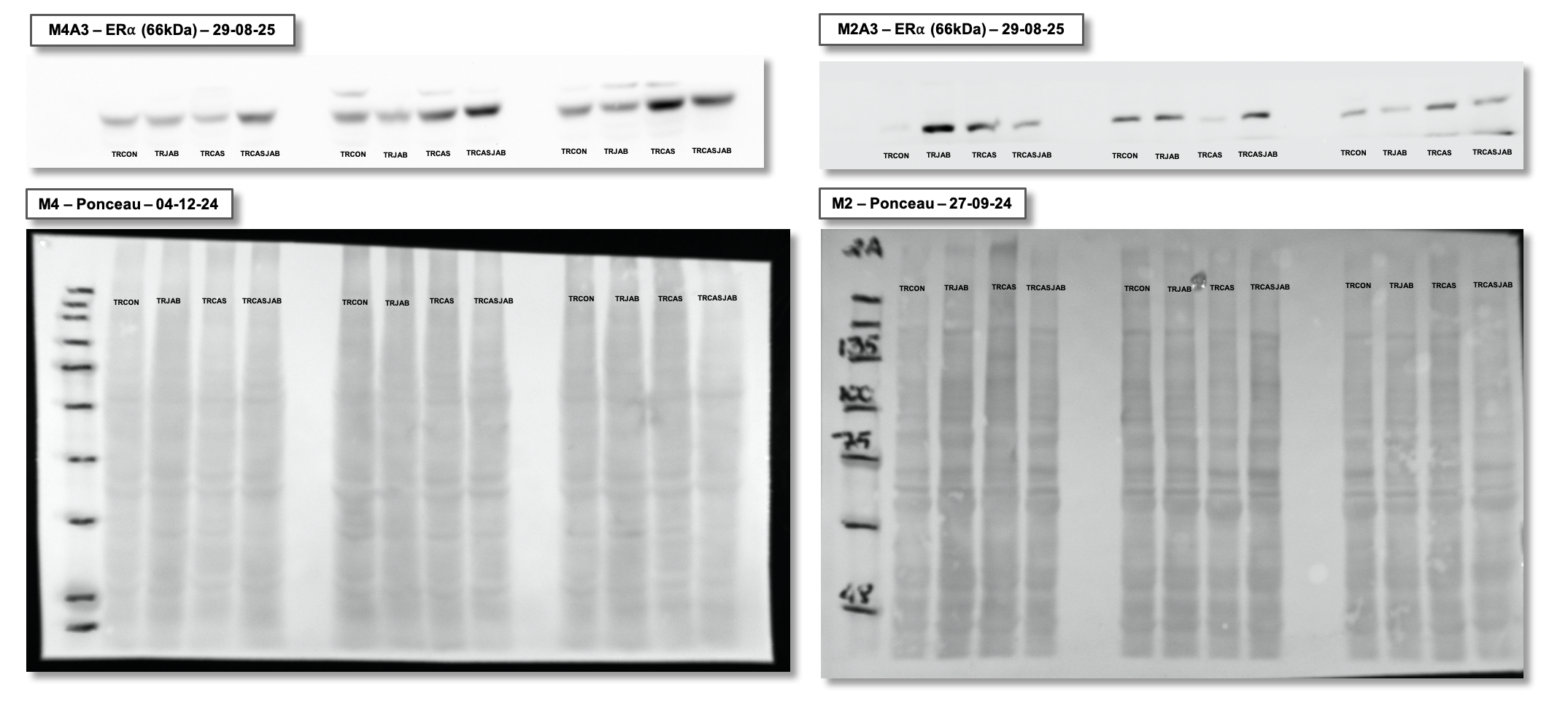


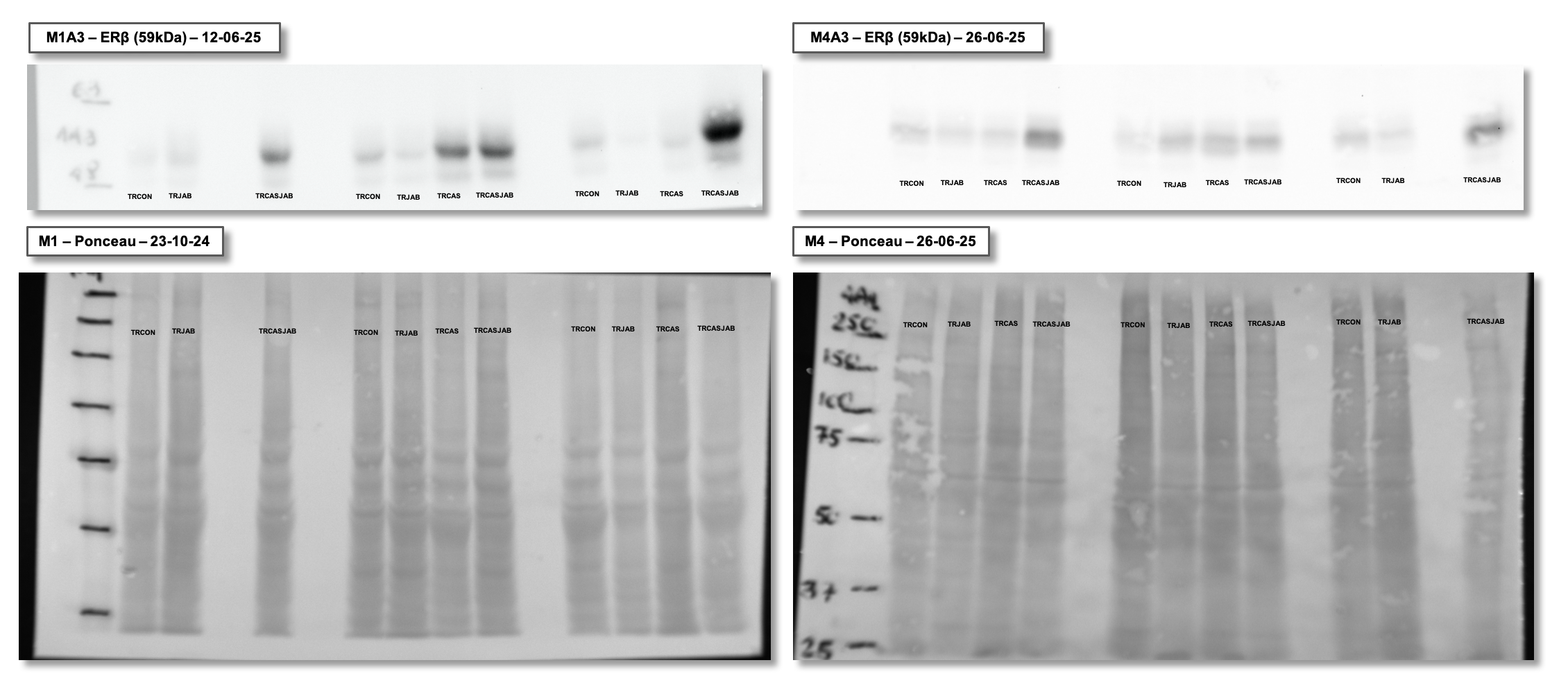


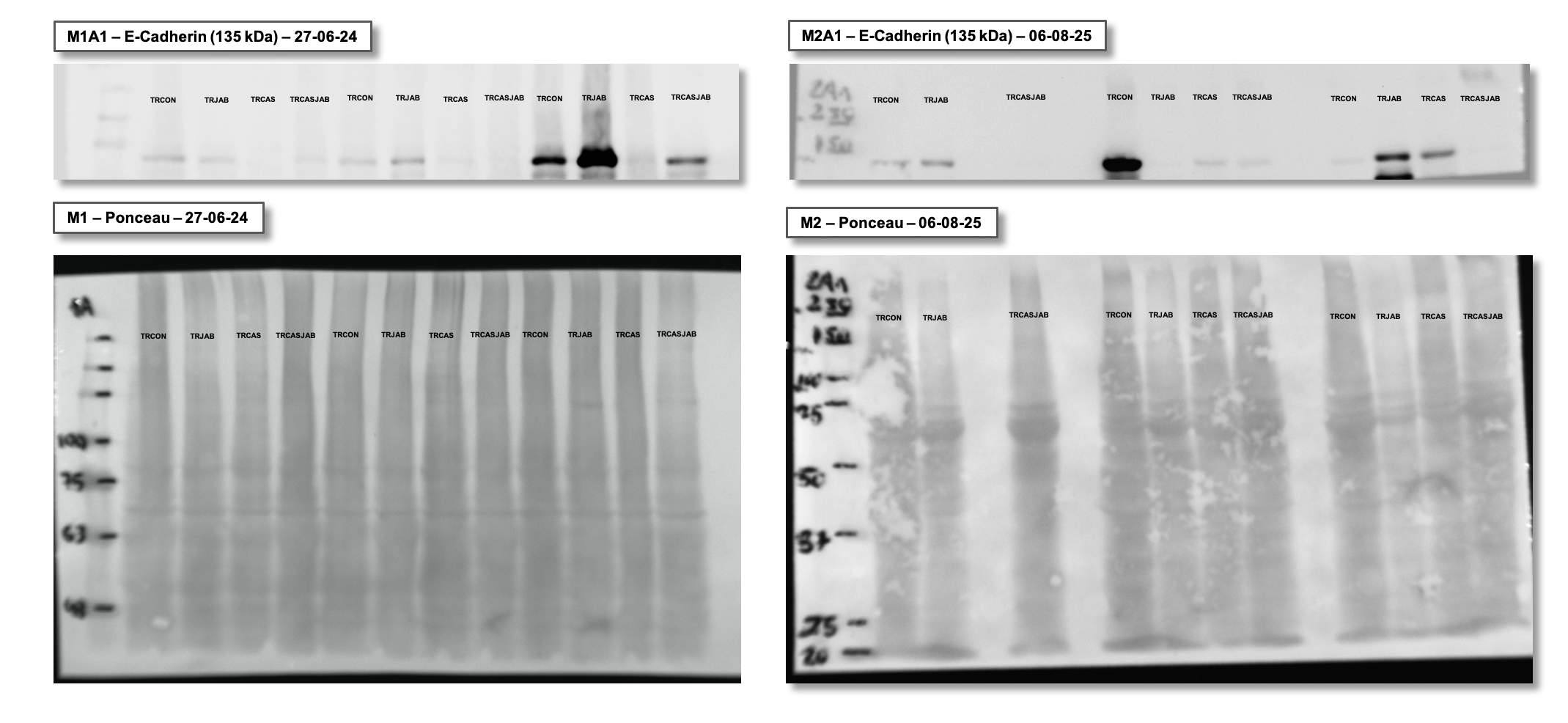


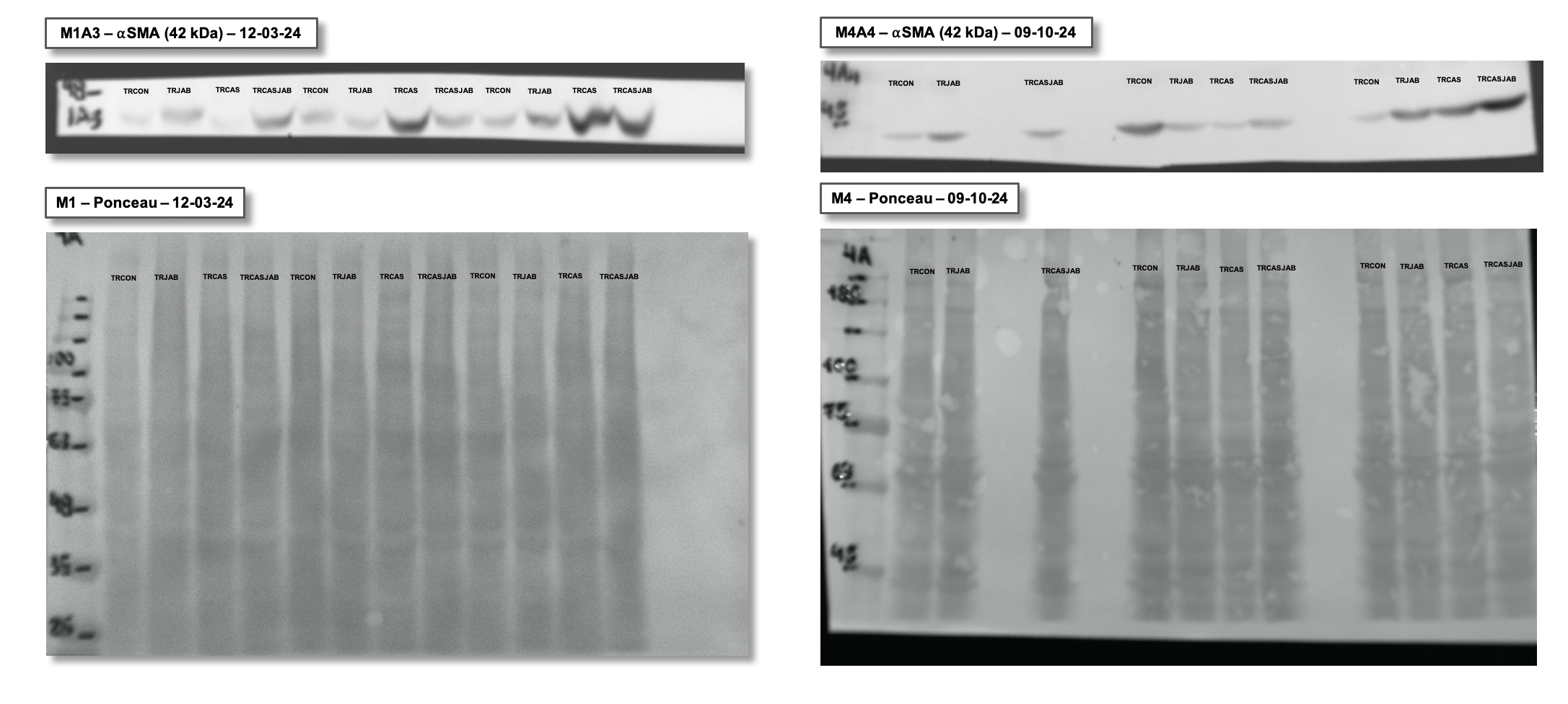


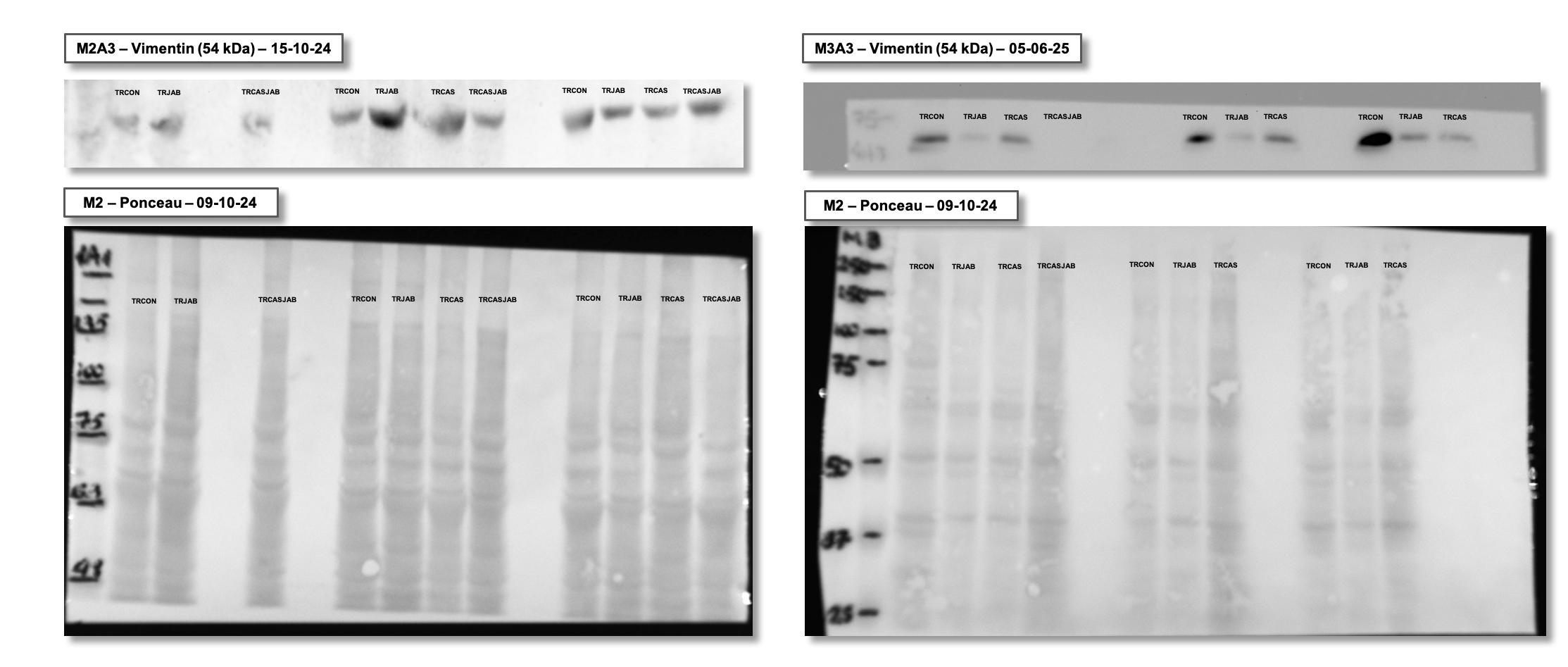


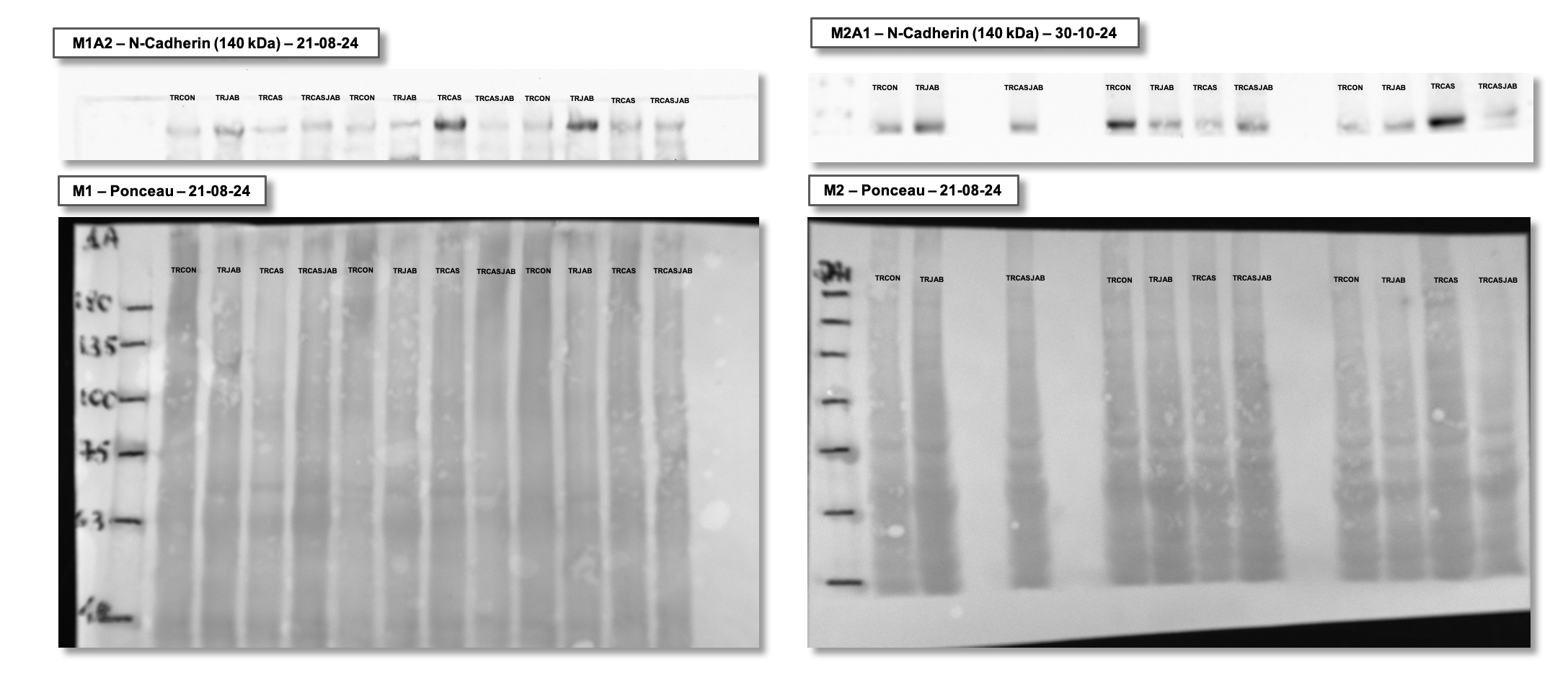


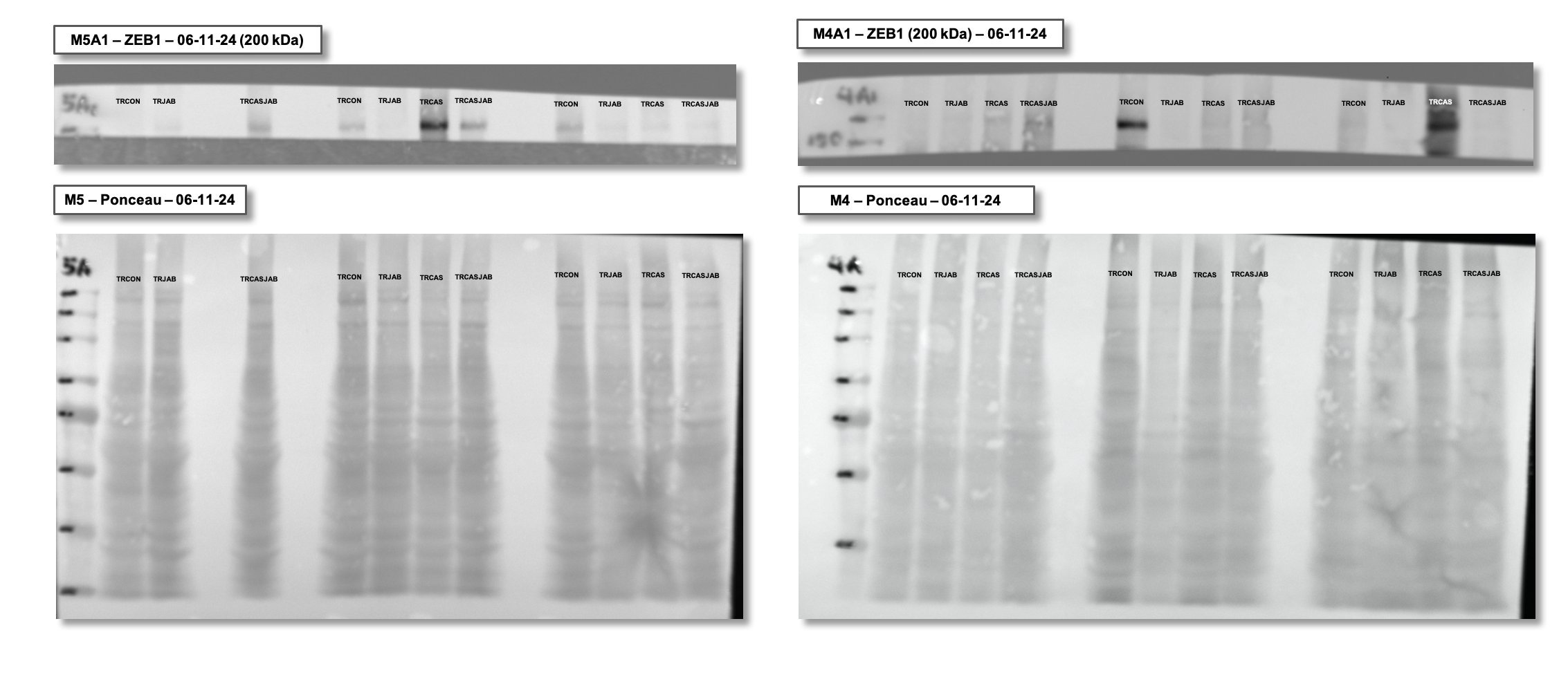


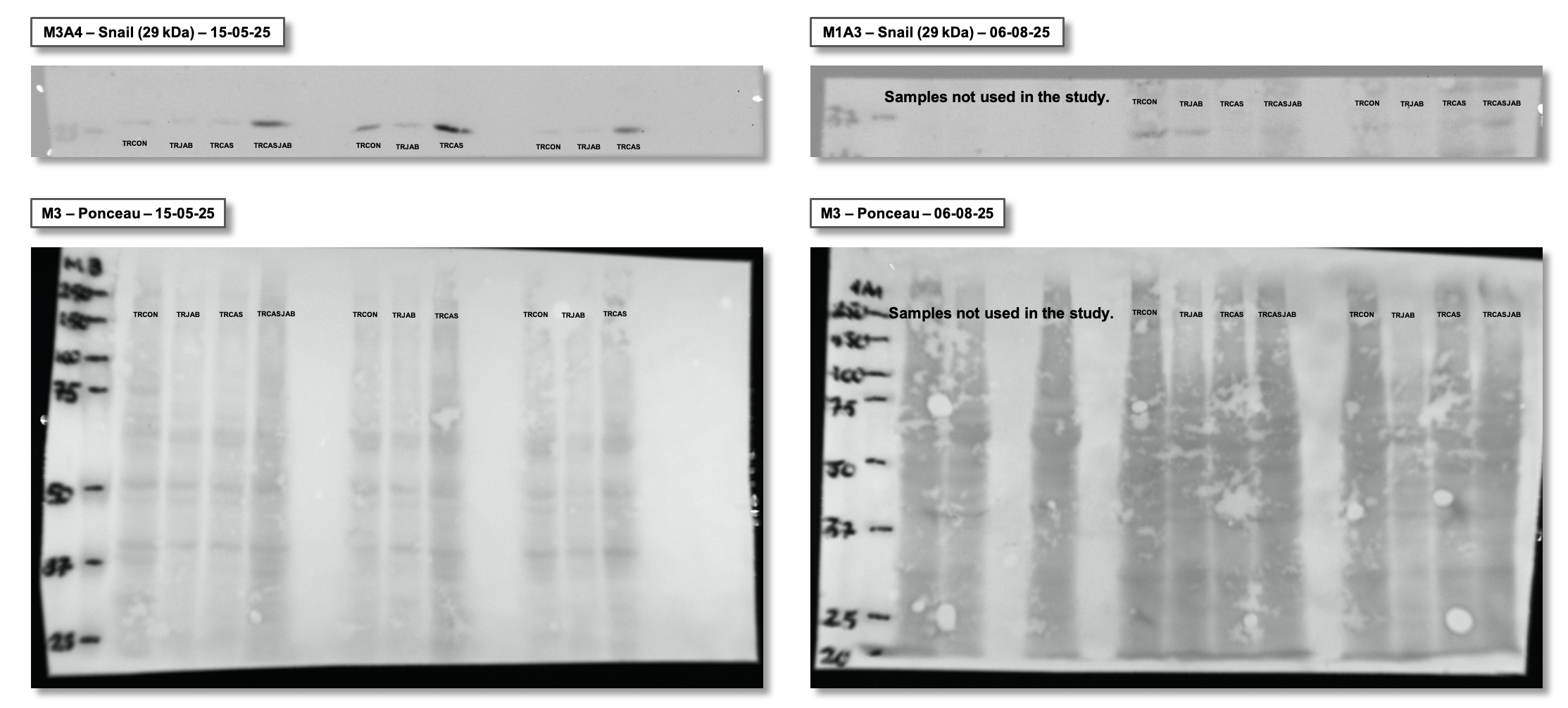


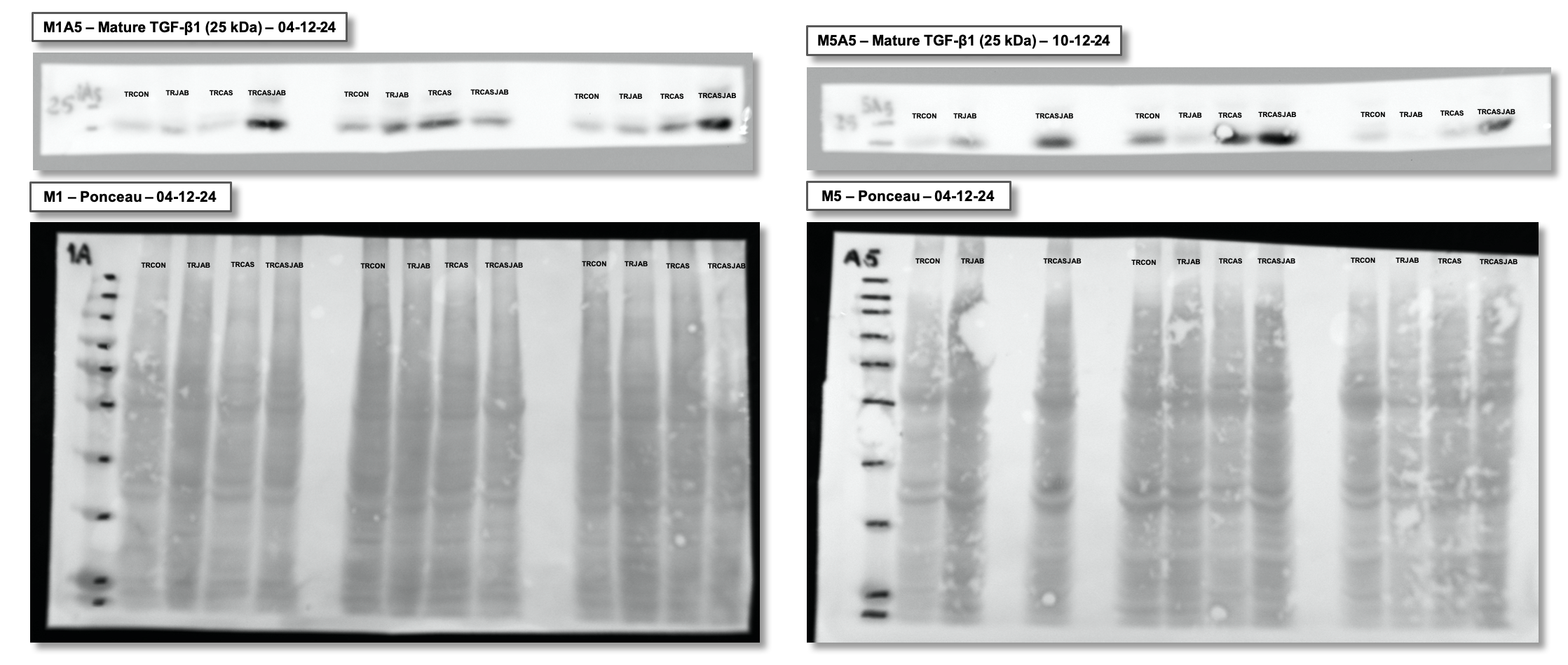


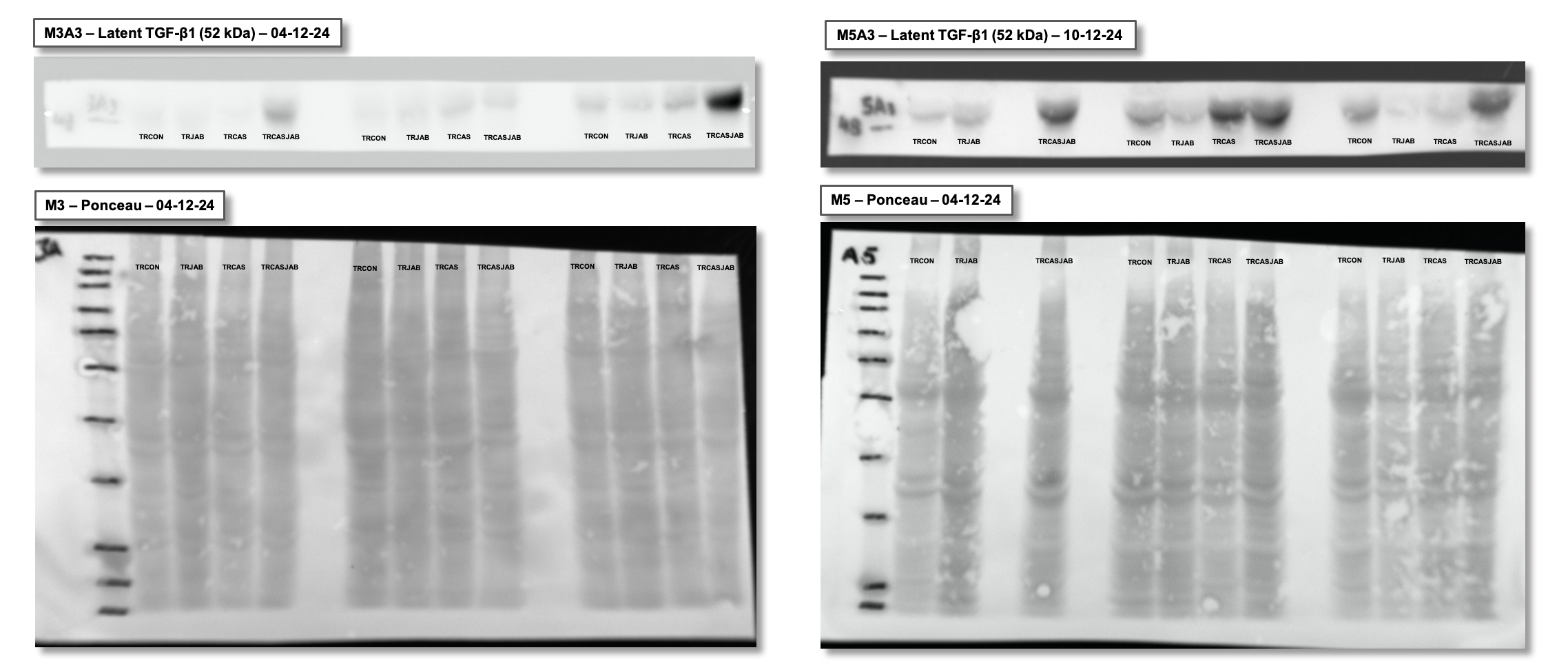


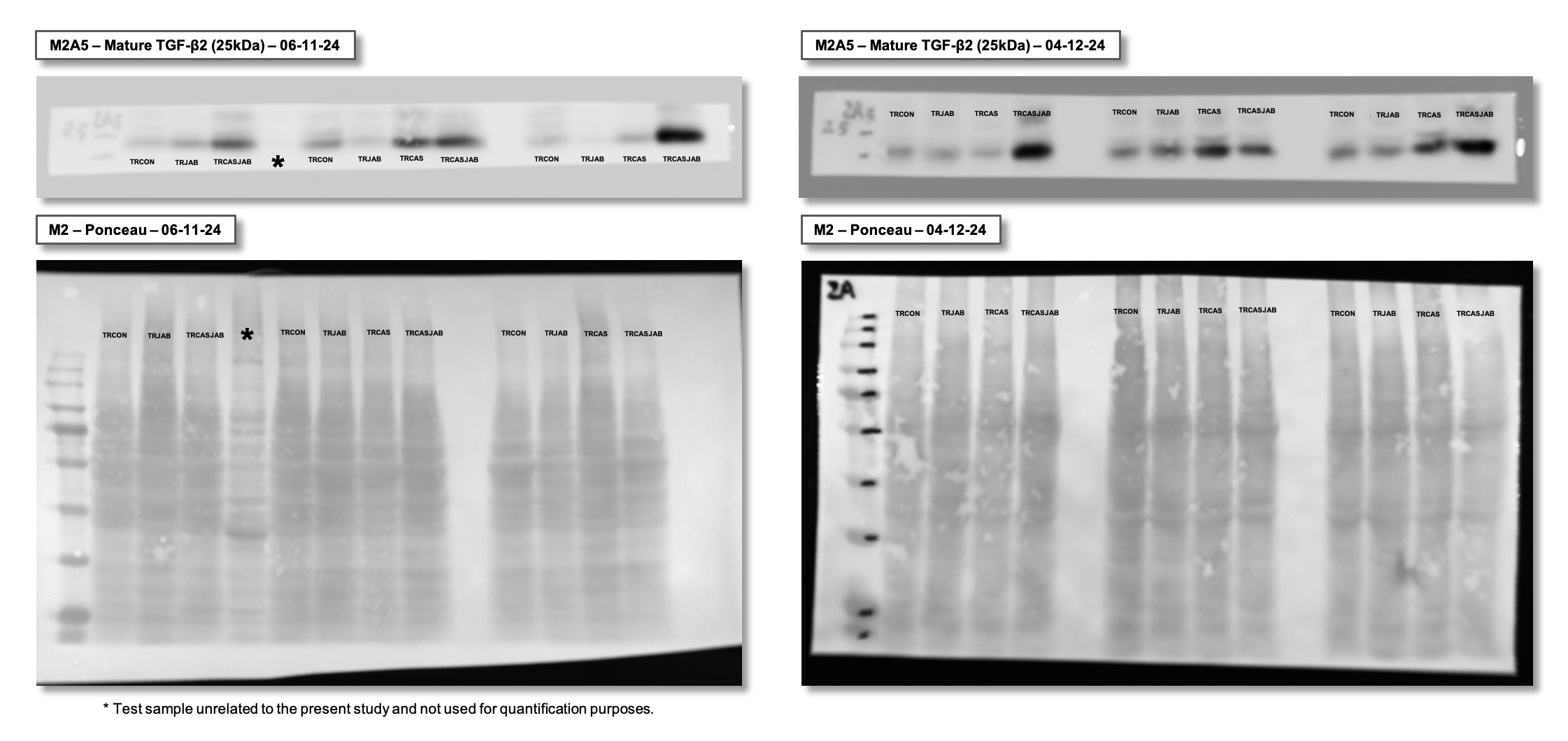


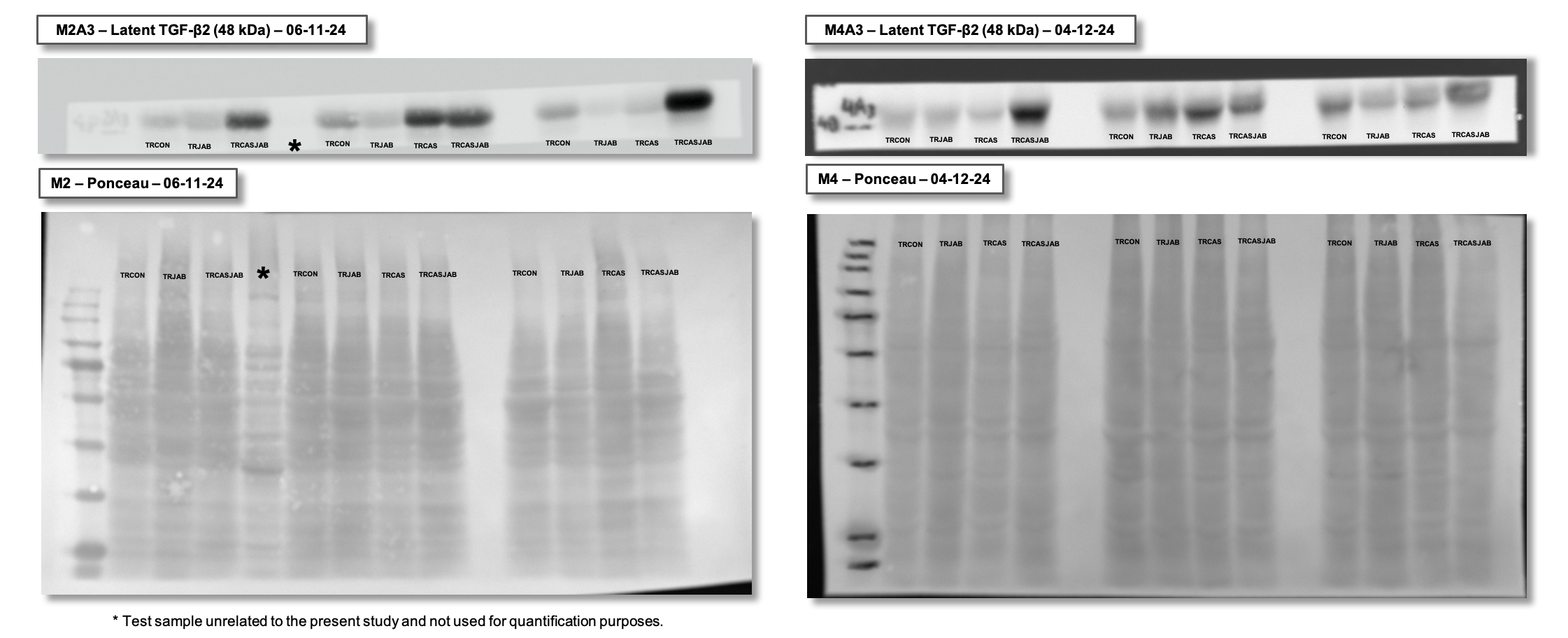


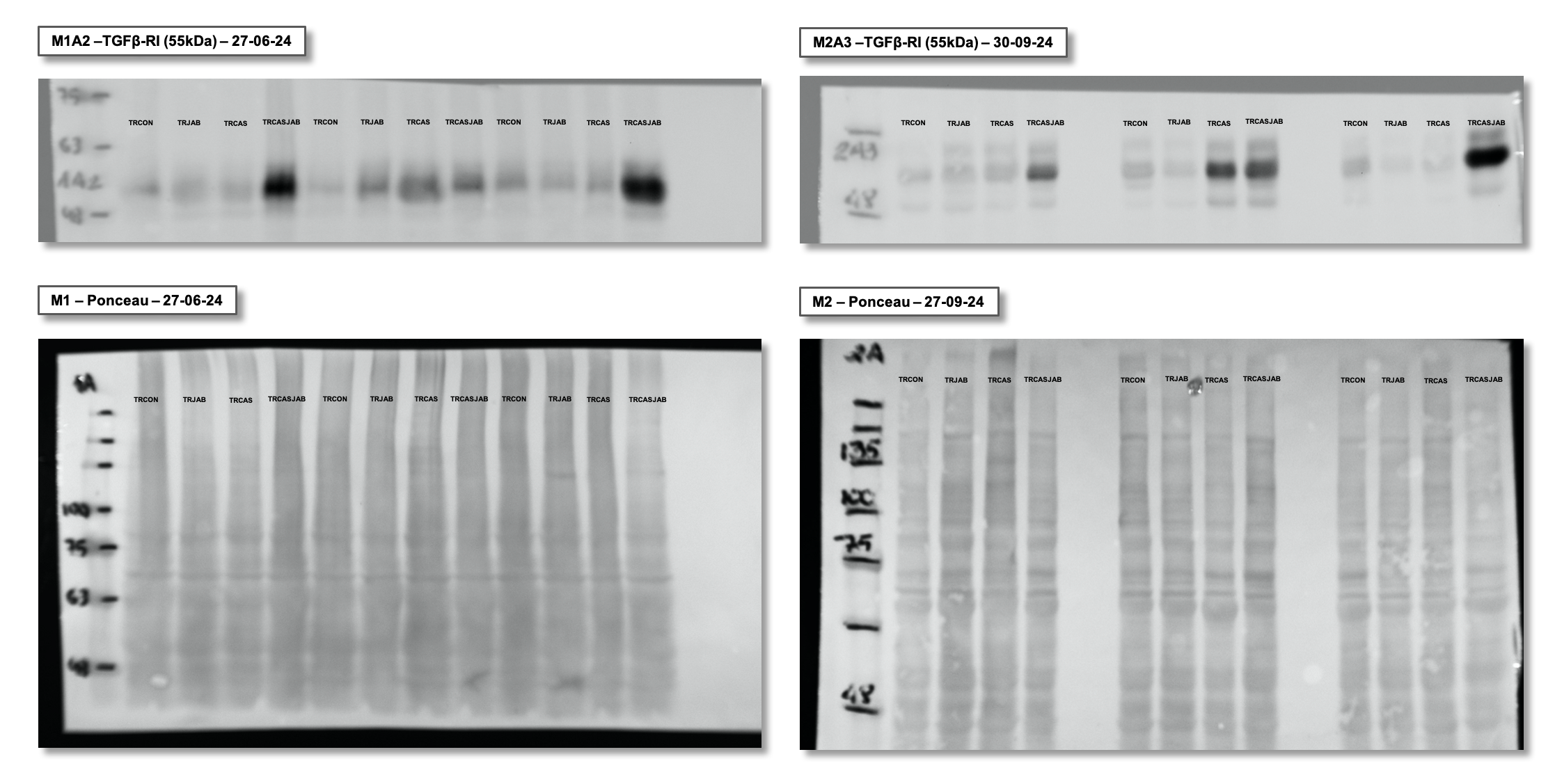


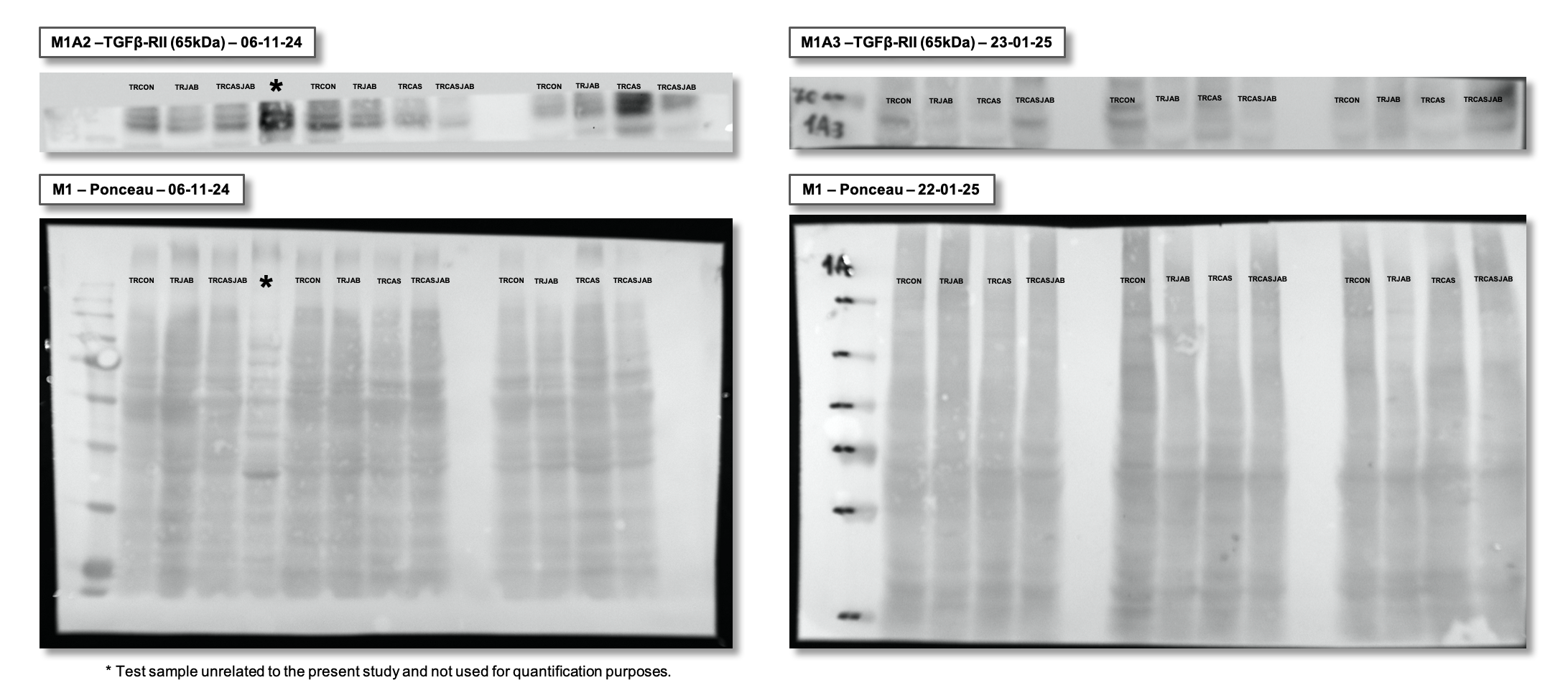

Supplement: Supplementary file 1 — Supplementary Material 1 [file 10735_2026_10851_MOESM1_ESM.docx]
